# Integration of human stem cell-derived *in vitro* systems and mouse preclinical models identifies complex pathophysiologic mechanisms in retinal dystrophy

**DOI:** 10.3389/fcell.2023.1252547

**Published:** 2023-08-24

**Authors:** Melissa K. Jones, Luz D. Orozco, Han Qin, Tom Truong, Patrick Caplazi, Justin Elstrott, Zora Modrusan, Shawnta Y. Chaney, Marion Jeanne

**Affiliations:** ^1^ Department of Neuroscience, Genentech Inc., South San Francisco, CA, United States; ^2^ Product Development Clinical Science Ophthalmology, Genentech Inc., South San Francisco, CA, United States; ^3^ Department of Bioinformatics, Genentech Inc., South San Francisco, CA, United States; ^4^ Department of Translational Immunology, Genentech Inc., South San Francisco, CA, United States; ^5^ Department of Research Pathology, Genentech Inc., South San Francisco, CA, United States; ^6^ Department of Translational Imaging, Genentech Inc., South San Francisco, CA, United States; ^7^ Department of Microchemistry, Proteomics, Lipidomics and Next-Generation Sequencing, Genentech Inc., South San Francisco, CA, United States

**Keywords:** retinal dystrophy, human stem cells, retinal organoids, mouse models, single cell sequencing, *DRAM2*, disease modeling

## Abstract

Rare *DRAM2* coding variants cause retinal dystrophy with early macular involvement via unknown mechanisms. We found that *DRAM2* is ubiquitously expressed in the human eye and expression changes were observed in eyes with more common maculopathy such as Age-related Macular Degeneration (AMD). To gain insights into pathogenicity of DRAM2-related retinopathy, we used a combination of *in vitro* and *in vivo* models. We found that *DRAM2* loss in human pluripotent stem cell (hPSC)-derived retinal organoids caused the presence of additional mesenchymal cells. Interestingly, *Dram2* loss in mice also caused increased proliferation of cells from the choroid *in vitro* and exacerbated choroidal neovascular lesions *in vivo*. Furthermore, we observed that *DRAM2* loss in human retinal pigment epithelial (RPE) cells resulted in increased susceptibility to stress-induced cell death *in vitro* and that *Dram2* loss in mice caused age-related photoreceptor degeneration. This highlights the complexity of DRAM2 function, as its loss in choroidal cells provided a proliferative advantage, whereas its loss in post-mitotic cells, such as photoreceptor and RPE cells, increased degeneration susceptibility. Different models such as human pluripotent stem cell-derived systems and mice can be leveraged to study and model human retinal dystrophies; however, cell type and species-specific expression must be taken into account when selecting relevant systems.

## 1 Introduction

Inherited retinal dystrophy (IRD) is a broad category of phenotypically and genetically heterogeneous disorders that affects about 1 in 3,000 people and is characterized by progressive and irreversible vision loss (Sahel et al., 2015). Sub-classifications of IRDs include further categorization into cell- or region-specific loss, including rod or cone/rod dystrophies and macular dystrophies, with the underlying pathogenesis ultimately due to photoreceptor degeneration. Mutations in greater than 270 genes have been identified as causative factors leading to IRDs (Bohórquez et al., 2021), with mutations in over 80 genes for the most common form, retinitis pigmentosa (RP) (Bravo-Gil et al., 2017). Luxturna^®^ (voretigene neparvovec-ryzl; Spark Therapeutics, Inc.) was the first FDA-approved gene therapy to treat patients with confirmed biallelic *RPE65* mutation-associated retinal dystrophy (Bennett et al., 2016; Maguire et al., 2019). This first approved pharmacologic treatment for IRDs caused by RPE65 mutations constituted a breakthrough therapy, and other therapies for a range of rare IRDs are now under clinical assessment (Prado et al., 2020).

The decreasing cost of emerging technologies, such as genome sequencing for personalized medicine, is changing the way patient care is approached, with treatment plans being increasingly tailored to each individual. Identification of pathologic mechanisms associated with genetic variations is therefore of foremost importance as it guides medical decisions and development of new therapeutic approaches. Recent technological advances in biomedical science, such as single cell sequencing, have allowed a deeper dive into tissue composition and disease pathophysiology (Tang et al., 2019). Even though in-depth molecular and cellular compositions of retinas from different species have been established (Shekhar et al., 2016; Rheaume et al., 2018; Liang et al., 2019; Peng et al., 2019; Yan et al., 2020a; 2020b; Lu et al., 2020; Orozco et al., 2020; Yamagata et al., 2021), constructing atlases of human disease such as IRDs remains challenging, as samples are rare, accessible only post-mortem, and usually not accompanied by a precise clinical diagnosis. Therefore, animal models are commonly used to model and study retinal diseases (Pennesi et al., 2012; Winkler et al., 2020). The development of *in vitro* human pluripotent stem cell (hPSC)-derived systems relevant to the eye, such as retinal organoids, has also provided researchers with new tools to study human-specific mechanisms, as they recapitulate retinal development and are comparable to human fetal retinal tissue (Cowan et al., 2020; Sridhar et al., 2020). Additionally, human induced pluripotent stem cell (iPSC)-derived retinal organoids generated from IRD patient cells have been used for *in vitro* modeling (Deng et al., 2018; Guo et al., 2019; Li et al., 2019; Lukovic et al., 2020; Kruczek et al., 2021). Of course, both animal models and human stem cell-derived *in vitro* systems have limitations, and as negative results are rarely published, it is hard to evaluate how successful they are at recapitulating aspects of particular IRDs.

*DRAM2*-associated retinopathy, also called cone-rod dystrophy 21 (CORD21), is a rare autosomal recessive IRD caused by coding variants in the *DNA-damage Regulated Autophagy Modulator 2* (*DRAM2*) gene (El-Asrag et al., 2015; Sergouniotis et al., 2015; Birtel et al., 2018; Abad-Morales et al., 2019; Kuniyoshi et al., 2020; Krašovec et al., 2022). *DRAM2*-retinopathy patients are usually asymptomatic in the first 2 decades of life, but then develop progressive central vision loss, associated with characteristic clinical features such as fine white/yellow dots, well-defined atrophic areas in the central retina, and bone-spicule pigmentation in the periphery. Following early maculopathy, visual acuity loss progresses and peripheral retinal degeneration is usually present in the later stages of the disease (El-Asrag et al., 2015; Krašovec et al., 2022).

*DRAM2*, also known as *Transmembrane protein 77* (*TMEM77*), encodes a 266-amino acid protein with six putative transmembrane domains localized in lysosomes (O’Prey et al., 2009; Park et al., 2009). DRAM2 was named after its homologue DRAM1, a key autophagy modulator and p53-cell death regulator (Crighton et al., 2007; O’Prey et al., 2009); however, DRAM2 cellular function remains unclear and controversial. DRAM2 has been involved in cell death (Park et al., 2009; Bai et al., 2016; Wudu et al., 2019), autophagy (Yoon et al., 2012) and more recently inflammation (Li et al., 2020; Liu et al., 2020). Of note, DRAM2 cellular function has often been studied in the context of oncogenicity and tumor cell treatment response, and not in the context of neurodegeneration or retinal dystrophy.

The purpose of our study was to use *DRAM2* loss as genetic perturbation and compare *in vitro* human and *in vivo* mouse models to determine if they could be leveraged to decipher pathophysiologic mechanisms of complex retinal dystrophy.

## 2 Materials and methods

### 2.1 Animal maintenance and ocular examination

All animal experiments were approved by the Genentech Institutional Animal Care and Use Committee (IACUC) and comply with the Institute for Lab Animals’ guidelines for the humane care and use of laboratory animals. Animals were housed with *ad libitum* access to water and food and on a 14 h light/10 h dark cycle except for the animals subjected to the constant light exposure (CLE) model. Both males and females were used for experiments, with the exception of the animals used for single cell RNA sequencing and sodium iodate model, for which only males were used.

For ocular examination (fundus imaging, fluorescein angiography, optical coherence tomography (OCT) scanning and electroretinography (ERG) recording), mice were anesthetized by intraperitoneal injection of ketamine (70–80 mg/kg body weight) and xylazine (15 mg/kg body weight). Pupils were dilated with drops of Tropicamide Ophthalmic Solution USP 1% (Akorn). Drops of Systane lubricant eye drop (Alcon) were applied bilaterally to prevent corneal dehydration during the procedure. After ocular examination, anesthetized mice were placed on a pre-warmed warming plate at 37°C until they awakened.

Fundus images were obtained using the Spectralis HRA + OCT system (Heidelberg Engineering). To adjust for rodent optics, the system was modified according to the manufacturer’s recommendations with a 55-degree wide-field lens placed in front of the camera.

Fluorescein angiography was performed with the Spectralis HRA + OCT system (Heidelberg Engineering). After anesthesia, mice were intraperitoneally injected with fluorescein AK-FLUOR (Akorn) at 5 μg/g body weight in physiological saline. Animals were orientated on a stage so that the optic nerve would be visible in the same location in each image and images acquired with a 488 nm light filter at 5- and 10-min post fluorescein injection.

Retinal thickness was measured by OCT scans using a Bioptigen Envisu R machine (Leica Microsystems, IL, United States). Total retina thickness was defined as the width from the nerve fiber layer to the RPE/choroid layer on the cross-sectional images. Retinal segmentation was automatically determined using an algorithm (Matlab software, MathWorks).

ERG recordings were performed with the Celeris electrophysiology system (Diagnosys). Mice were dark-adapted overnight before ERG recording, and all procedures were performed under low-level red light. Mouse body temperature was maintained on a 37°C homeothermic plate. A reference electrode was inserted subcutaneously through the forehead and a ground electrode was inserted subcutaneously at the base of the tail. Electrodes with a light-stimulator were placed on both eyes. Under scotopic conditions, eyes were stimulated with six flash-intensities at 0.001, 0.01, 0.05, 0.1, 2 and 4 cd s/m^2^. After 5 min of light adaptation, eyes were then stimulated with photopic flash of 4 intensities at 2, 5, 50 and 250 cd.S/m^2^. Recorded signals were bandpass-filtered at 0.15–1,000 Hz and sampled at 2 kHz. All of the recorded data points were analyzed using a custom Matlab software (MathWorks) with a-wave amplitude measured from the baseline to the trough of the a-wave while b-wave amplitude from the trough of the a-wave to the peak of the b-wave. Responses to three to five flashes of light stimulation were averaged.

### 2.2 Mouse ocular pre-clinical models

For the constant light exposure (CLE) degeneration model, animals were housed in transparent plastic boxes and exposed to 100,000 lux of white LED lighting (measured using an Extech HD450 light meter) for 24 h per day for 7 days. Each animal was administered with pupil dilator eye-drops twice daily (Atropine Sulphate 1% Akorn).

For the sodium iodate (NaIO_3_) degeneration model, male mice were intravenously injected with 20 mg/kg body weight of NaIO_3_ (Sigma-Aldrich) or saline control.

For the laser-induced choroidal neovascularization (CNV) injury model, animals received analgesic (buprenorphine, 0.05 mg/kg) intraperitoneally the day of the procedure. Mice were then anesthetized by intraperitoneal injection of ketamine (70–80 mg/kg body weight) and xylazine (15 mg/kg body weight). Pupils were dilated with drops of Tropicamide Ophthalmic Solution USP 1% (Akorn). Neovascularization was induced in each eye using an image-guided laser system (Micron III, Phoenix Research Laboratories) with a 532 nm wavelength (laser spot size of 100 μm, 320 mW power and 80 m duration). Three burns in eye at the 3, 6, and 12 o’clock positions around the optic nerve were made, and each burn was made 2–3 optic disk diameters (about 200–300 μm) from the optic nerve. Cases of hemorrhage induced by the laser were excluded from the analysis. After the procedure, a topical antibiotic (Neomycin and Polymyxin B Sulfates and Bacitracin Zinc Ophthalmic Ointment, Bausch & Lomb) was applied to both eyes and the mice were placed on a pre-warmed warming plate at 37°C until they awakened. CNV lesions were outlined and the surface area of each lesion was quantified using the Imaris software.

### 2.3 Histopathology, *in situ* hybridization and immunofluorescence

*Postmortem* human donor eyes were obtained from the Lions Eye Institute for Transplant and Research in Tampa, Florida. After 24-h fixation in 10% neutral-buffered formalin (NBF), both human and mouse eyes were transferred into 70% ethanol until paraffin embedding in an automated paraffin tissue processor. The formalin fixed paraffin embedded (FFPE) sections were subjected to deparaffinization before further processing. Hematoxylin-eosin (H&E) staining was performed according to standard protocol using an Automated Slide Stainer.

The *in situ* hybridization (ISH) RNAScope™ 2.5 HD-RED manual assay (Advanced Cell Diagnostics (ACD)) was performed on 4 μm-thick formalin-fixed paraffin-embedded sections of human or mouse eyes according to the ACD protocol. Probes against the ubiquitously expressed isomerase PPIB were used as positive control, and probes against bacterial DapB were used as negative control. ISH on mouse eye sections were performed using a 20 ZZ probe targeting 263-1479 of NM_001025582.2, and the ISH on human eye sections were performed using a 20 ZZ probe targeting 369-1475 of NM_001349881.1. All the probes were provided by ACD. After deparaffinization in xylene and endogenous peroxidase activity inhibition by H_2_O_2_ (10 min), sections were permeabilized and submitted to heat (15 min at 100°C) and protease IV treatment (20 min at 40°C). After probe hybridization for 2 h at 40°C, the signal was chemically amplified using the kit reagents and detected using the FastRED dye. The sections were then counterstained with Hematoxylin and mounted using VectaMount (Vector Labs, H-5000).

For immunofluorescent labeling, cells or retinal organoid sections were fixed in 4% paraformaldehyde and blocked/permeabilized with 10% normal donkey serum/0.1% Triton X-100/PBS. Cells or sections were incubated for 2 h with primary antibodies against: Zonula occludens-1 (ZO-1) (1:100; 61-7300, ThermoFisher), Melanocyte inducing transcription factor (MiTF) (1:200; ab12039, Abcam), Visual system homeobox (CHX10/VSX2) (1:200; SC-365519, Santa Cruz), Orthodenticle homeobox 2 (OTX2) (1:200; AB9566-I, Milipore), Rhodopsin (1:200; SC-57432, Santa Cruz), Arrestin 3 (ARR3) (1:200; AB15282, Millipore), Matrix Gla protein (MGP) (1:500; MA5-26799, Invitrogen), and Decorin (DCN) (1:100; AF143, R&D). Respective secondary antibodies were conjugated to either Alexa Fluor 488 or Alexa Fluor 594 (Invitrogen) and used at 1:500. Sections were mounted using Mowiol containing DAPI for nucleus staining.

### 2.4 Cell culture, treatments and functional assays

Human primary retinal pigment epithelial (hRPE) cells (Lonza #00194987) were maintained in Retinal Pigment Epithelial Cell Growth Medium (RtEGM; Lonza) per manufacturer protocols.

The pluripotent human embryonic stem cell (hPSC) line H1 was purchased from WiCell (WA01, (Thomson et al., 1998)) and experiments were performed prior to passage 40. hPSCs were maintained on growth factor reduced Matrigel (BD Biosciences) coated plates with mTeSR1 medium (Stemcell Technologies) according to WiCell protocols. Cells were passaged every 5–7 days at 80% confluence using Gentle Cell Dissociation Reagent (Stemcell Technologies). Colonies containing clearly visible differentiated cells were mechanically removed before passaging.

For isolation and culture of mouse RPE/choroid cells, mouse eyes were enucleated and the anterior chamber, the lens and the neural retina were removed. The RPE/choroid were then dissociated with papain solution (40 U papain in 10 mL DPBS) for 45 min at 37°C. Papain was neutralized in a trypsin inhibitor solution (0.15% ovomucoid in DPBS), and the tissue was triturated to dissociated cells. Following trituration, the cells were pelleted, resuspended, and cultured in RtEGM for experiments.

For cell confluency and cell survival assays (using the cell-impermeable DNA-binding dye DRAQ7), cells were imaged using the Incucyte S3 (Essen BioScience) and quantified using the Incucyte Cell-by-Cell Analysis Software Module. For the *in vitro* degeneration pre-clinical models, sodium iodate (NaIO_3_) at 5 mM or *N*-retinylidene-*N*-retinylethanolamine (A2E) at 30 µM was added to the culture medium.

To conjugate fluorescence to the outer segments, bovine rod photoreceptor outer segments (POS; Invision BioResources) were labeled with FITC Isomer I (Sigma-Aldrich) per published protocol (Parinot et al., 2014). Briefly, FITC Isomer I was reconstituted in carbonate buffer (final concentration 2.5 mg/mL) and rotated for 1 h at room temperature (RT) in the dark. POS from 50 eyes (Lot #bROS-210820) were resuspended in DMEM, FITC was added, and rotated for 1.5 h at RT in the dark. FITC labeled POS (FITC-POS) were washed 4x with DMEM, centrifuged, and resuspended. The total number of particles were counted and diluted to 8 x 10^7^ particles/mL for storage at −80°C until use. To perform the phagocytosis assay, FITC-POS (approximately 8 × 10^6^ particles) were fed to the RPE cells for 6 h in the dark at 37°C (*n* = 4 transwells/replicate, n = 3 technical replicates). Following incubation, cells were washed 3x with RPE maintenance media (RPEMM) and either fixed in 4% PFA for 10 min at RT for ICC or prepared for flow cytometry analysis. To quantify the FITC-POS phagocytized, flow cytometry was performed (BD FACSymphony S6). Following washing with RPEMM, RPE cells were dissociated for 10 min in trypsin-EDTA, collected, filtered in a 35 μm cell strainer, and stored in cold FACS buffer (1X PBS, 0.5% BSA, 0.05% sodium azide).

### 2.5 Genetic CRISPR/Cas9 knockout and lentiviral knockdown

*Dram2* knockout mice were generated by targeted mutation using CRISPR/Cas9 technology to delete exon 4. The resulting allele was named *Dram2* ^*tm1Jean*^ in accordance with the guidelines from the International Committee on Standardized Genetic Nomenclature for Mice. To simplify our notations, the functionally null *Dram2* ^*tm1Jean*^ allele is denoted knockout (ko) hereafter. Electroporation-based strategy of C57BL/6J zygotes with 25 ng μL^−1^ wild-type Cas9 mRNA (Life Technologies) and 13 ng μL^−1^ *in vitro*-transcribed two single-guide RNA into mouse zygotes was used (Modzelewski et al., 2018). Target sequences of sgRNA used to knockout exon4 were 5′- AGC​ATA​CTG​TTA​GCA​AAT​CA (PAM: AGG, CFD algorithm score of 92) and 5′-TTA​TCT​AAA​CCT​AAG​TTG​CA (PAM:AGG, CFD algorithm score of 84). The 585 bp knockout region corresponds to GRCm38/mm10 chr3: 106,561,518- 106,562,102. Tail DNA from resulting offspring was analyzed by PCR and sequencing. Genotyping was carried out using the following primers: 5′-CTA​AGA​CAA​TAA​CTG​ATG​AAT​GGT, 5′-AGC​GAG​CAA​GAG​AAC​ATA​A, and 5′-ACA​CAC​AAG​ACA​GGA​ACT​T.

For generation of the *DRAM2* knockout (KO1 and KO2) hPSC lines, a guide RNA (gRNA) targeting *DRAM2* exon3 (5′-AAG​GTA​AAG​CCG​GGT​CTA​TA) was designed and generated using GeneArt Precision gRNA Synthesis Kit (Invitrogen). hPSCs were trypsinized to single cells and electroporated with gRNA and rCas9 protein (ThermoFisher) using Human Amaxa P3 Primary Cell 96-well Nucleofector Kit on 4D-nucleofector X unit with program CB-150 according to the manufacturer’s protocols (Lonza). After electroporation, cells were plated onto Matrigel coated cell culture plates with mTeSR1 PLUS medium in the presence of the 10 μM ROCK inhibitor Y-27632 (Selleckchem). After 10 days, single colonies were picked and expanded. To screen clones, genomic DNA was isolated using the Puregene Cell Kit (Qiagen) then PCR amplified with High-Fidelity 2X PCR Master Mix (NEB) using two primers flanking the target region (5′-ACT​TCG​TAC​GCA​GTA​AGC’ and 5′-GGC​TAA​AGT​AGG​ATG​AGG). PCR products were cloned into a T-vector (Promega), and sequenced. Two different hPSC clones homozygous for *DRAM2* knockout (KO1 and KO2) were selected for experiments.

For gene knockdown, shRNAs targeting *DRAM2* in lentiviral vector pGIPZ-CMV-tGFP-IRES-puro were purchased from Dharmacon (RHS4430-200179097 and RHS4430-200259023). To produce lentiviruses, HEK 293T cells at 60%–70% confluency were transfected in 10 cm plates with 5 μg of the lentiviral vectors together with packaging plasmids 3.5 μg delta8.9 and 1.7 μg VSVG using Lipofectamine 2000 (ThermoFisher). After 72 h, viral supernatants were harvested, filtered, titered, and stored at −80°C. Cells were infected in the culture medium in presence of virus for 48 h. *DRAM2* knockdown was confirmed by qRT-PCR. RNA was isolated using the RNeasy Plus Mini RNA Isolation kit (QIAGEN) and reverse-transcribed using the High-Capacity cDNA Reverse Transcription kit (Applied BioSystems). The cDNA reaction was diluted 1:5 in TE (10 mM Tris-Cl/1 mM EDTA, pH 7.6) and used in SsoFast EvaGreen Supermix with Low ROX (BioRad). Reactions were run in triplicates on a ViiA 7 machine (Applied BioSystems) according to the manufacturer’s instructions. Values were normalized to the housekeeping gene expression GAPDH and then to expression in uninfected cells. Data are from triplicate PCR reactions, and error bars represent standard deviation. Primers used were: *DRAM2* 5′-TCA​GCA​AGG​CCT​CAG​TTT​CC and 5′-GTA​GCA​ATG​CAT​AAA​ACT​GCC​G; *GAPDH* 5′-CTG​CAC​CAC​CAA​CTG​CTT​AG’ and 5′-TTC​AGC​TCA​GGG​ATG​ACC​TT.

### 2.6 hPSC directed differentiation into RPE cells and retinal organoids

Retinal pigment epithelial (RPE) cells were differentiated from hPSCs per published protocol with some modifications (Maruotti et al., 2015). Briefly, hPSCs were seeded at high density (20,000 cells per cm^2^) on growth factor-reduced Matrigel (BD Biosciences)-coated plates with mTeSR1 medium in 5% CO_2_. Cells were maintained for 10 days until forming a monolayer. From days 11–25, cells were given differentiation media which included DMEM/F12, 15% knockout serum replacement (KOSR; Invitrogen), 2 mM glutamine, 0.1 mM NEAA (Invitrogen), and 10 mM nicotinamide. After day 25, cells were switched to maintenance media (RPEMM) containing DMEM, F12, and 2% B27 without vitamin A (Invitrogen). At day 35, RPE were passaged using Accumax (Sigma) incubated for 20 min at 37°C. Cells were centrifuged at 130 x g for 3 min, filtered through a 40-µm nylon mesh (BD Falcon) and counted. Cells were plated at a density of 300,000 cells per cm^2^ onto growth factor-reduced Matrigel (BD Biosciences)-coated plates or transwells (Corning).

Retinal organoids were differentiated from hPSCs based on a previously described protocol with modifications (Idelson et al., 2009; Meyer et al., 2009; Zhong et al., 2014; Gong et al., 2015). Briefly, hPSCs were detached by Gentle Cell Dissociation Reagent (Stemcell Technologies), dissociated into small clumps and cultured in suspension with mTeSR1 medium and 10 μM Y-27632 (Selleckchem) to induce aggregate formation. Aggregates were gradually transitioned into neural-induction medium (NIM) containing DMEM/F12 (1:1), 1% N2 supplement (Gibco), 1x non-essential amino acids (NEAA; Gibco), 2 μg/mL heparin (Sigma), by replacing the medium with a 3:1 ratio of mTeSR1/NIM on day 1, 1:1 on day 2% and 100% NIM on day 3. On day 7, aggregates were seeded onto Matrigel (growth factor-reduced; BD Biosciences) coated dishes containing NIM at an approximate density of 20 aggregates per cm^2^. Starting day 16, the media was switched to retinal differentiation medium (RDM) containing DMEM/F12 (3:1) supplemented with 2% B27 (minus vitamin A; Gibco), 1x NEAA and 1x penicillin and streptomycin and was changed daily. Around day 28, horseshoe-shaped neural retina domains were collected and cultured in RDM, where they gradually formed 3D eye organoids. Thereafter, the media was changed twice a week. To mature eye organoids, the medium was supplemented with 10% fetal bovine serum, 100 μM Taurine (Sigma) and 1x GlutaMAX starting from day 42. To promote photoreceptor maturation, the retinal organoids were supplemented with 1 μM all-trans retinoic acid (RA) from weeks 10–14. Subsequently, RA concentration was decreased to 0.5 μM, and B27 supplement was switched to N2 supplement. Retinal organoids were cultured for up to 12 months.

### 2.7 Single cell RNA sequencing and bioinformatic analysis

Mouse and retinal organoid single-cell suspensions were prepared by adapting previously described methods (Macosko et al., 2015). Briefly, mouse retinas, mouse RPE/choroid preparations, and hPSC-derived retinal organoids were digested in papain solution (40U papain in 10 mL DPBS) for 45 min at 37°C. Papain was then neutralized in a trypsin inhibitor solution (0.15% ovomucoid in DPBS) and the tissue was triturated to generate a single-cell suspension. Following trituration, the cells were pelleted, resuspended, and filtered through a 20 μm Nitex mesh filter to eliminate any clumped cells. The cells were then diluted in DPBS with 3% FBS to 200 to 1,000 cells/μL. The scRNAseq library was generated using Chromium Single Cell 3′ Reagent Kit v2 (10X Genomics) per manufacturer’s instructions. Human donor eye single-nuclei suspensions were prepared from frozen sections and used for library preparation (Chromium single cell 3′ kit v2 or v3, 10X Genomics) and nucSequencing as described in (Orozco et al., 2020).

Single-nuclei RNAseq data were processed using cellranger from 10X. Since RNA derived from nuclei for the human single-nucleus RNAseq dataset was used, both exonic and intronic reads were considered for downstream analysis by including introns in the pre-processing step of the human reference genome sequence. For the single-cell RNAseq datasets, only exons were in the step to pre-processing the genomes. For alignment, the human reference genome GRCh38, and the mouse reference genome mm10 were used. The algorithm outputs a count matrix of cells by genes, which was used for down-stream analysis, and the clustering and dimensionality reduction analysis that is output by cellranger was not utilized.

Further downstream analysis was performed using Seurat. For normalization, UMIs using the “LogNormalize” method was utilized, and integration of the cells using CCA using experiment “Batch” as the batching variable was performed. For dimensionality reduction, we selected variable genes based on dispersion, then used these to compute principal components and UMAP dimensional reductions. Clusters of transcriptionally related cells corresponding to cell types or cell subtypes by using the graph-based clustering Louvain algorithm were generated and implemented in the Seurat function “FindClusters.” Cluster markers, i.e., gene expression markers that were more highly expressed in each cluster relative to all other clusters, using the “FindAllMarkers” function were searched, based on the non-parametric Wilcoxon rank-sum test. Cluster marker genes were considered if they were expressed in at least 10% of the cells in the cluster, with a minimum difference of 30% in the fraction of cells expressing the marker between two clusters, and a minimum log2 fold change in expression of 0.25.

For pseudo-bulk differential expression analysis, pseudo-bulk datasets were derived from each dataset by aggregating the cells of each sample of the same cell type using “aggregateAcrossCells” in scran as described (McCarthy et al., 2017). For n donors and m cell types, it creates n*m total possible pseudo-bulks, which are aggregates of cells of a single cell type from a single donor. The resulting pseudo-bulk count matrix was then normalized to a normalized count statistic using “logNormCounts” in scran, and size factors were calculated using edgeR (Robinson et al., 2010). Differential expression was performed on this data-set to compare control *versus* DRAM2 KO samples, for each cell type, using the voom-limma method for bulk RNAseq as described in the following.

For bulk RNAseq differential expression analysis, sequencing data analysis was performed as previously described (Durinck et al., 2015). Briefly, sequencing reads were mapped to the reference human genome (GRCh38), using the GSNAP short read aligner (Wu and Nacu, 2010). Transcript models used for differential expression were based on GENCODE annotations. Expression counts per gene were quantified using HTSeqGenie (Pau and Reeder, 2022). Expression counts were normalized to a normalized count statistic using “logNormCounts” in scran, and size factors were estimated using edgeR. Differential expression between bulk RNAseq samples using linear modeling with the voom/limma package (Law et al., 2014), and adjusted *p*-values for multiple testing using the Benjamini–Hochberg method were performed. Genes were considered differentially expressed if they had adjusted *p*-value < 0.05 and absolute fold change > 2.

In all heatmaps, color is the Z score of the expression level scaled by rows.

All datasets are available in the NCBI gene expression omnibus (GEO) database:

Already published datasets from Orozco et al. (2020): Bulk RNA seq of retinas and RPE/choroids (GSE135092) and Single-nucleus RNA seq of human retinas (GSE135133).

New datasets generated during this study are accessible in the reference series GSE220627 at https://www.ncbi.nlm.nih.gov/geo/query/acc.cgi?acc=GSE220627.

It includes three subseries.

- the hPSC-derived retinal organoids scRNA sequencing data (GSE220624)
- the mouse RPE/Choroid scRNA sequencing data (GSE220625).
- the mouse retina scRNA sequencing data (GSE220626).

### 2.8 Statistical analysis and software

Statistical analyses were performed using GraphPad Prism 9. Means+/-standard deviation are shown on all graphs. Exact values of numbers of samples used are described in Results or Figure Legends. All figures were created with BioRender (BioRender.com).

### 2.9 Gene and disease references

Cone-rod dystrophy 21 (CORD21): MIM# 616502; *DNA-damage Regulated Autophagy Modulator 2* (*DRAM2*) gene: MIM# 613360; DRAM1 gene: MIM# 610776; *CNGB3* gene: MIM# 605080; achromatopsia 3: MIM# 262300; *POC1B* gene: MIM# 614783; cone-rod dystrophy associated with *POC1B* mutations: MIM#615973; *CRB1* gene: MIM# 604210; retinal dystrophies associated with *CRB1* mutations: MIM# 613835, 172870, 600105; *BEST1* gene: MIM# 607854.

## 3 Results

### 3.1 DRAM2 is ubiquitously expressed in the human eye and expression changes are associated with macular degeneration

**FIGURE 1:**
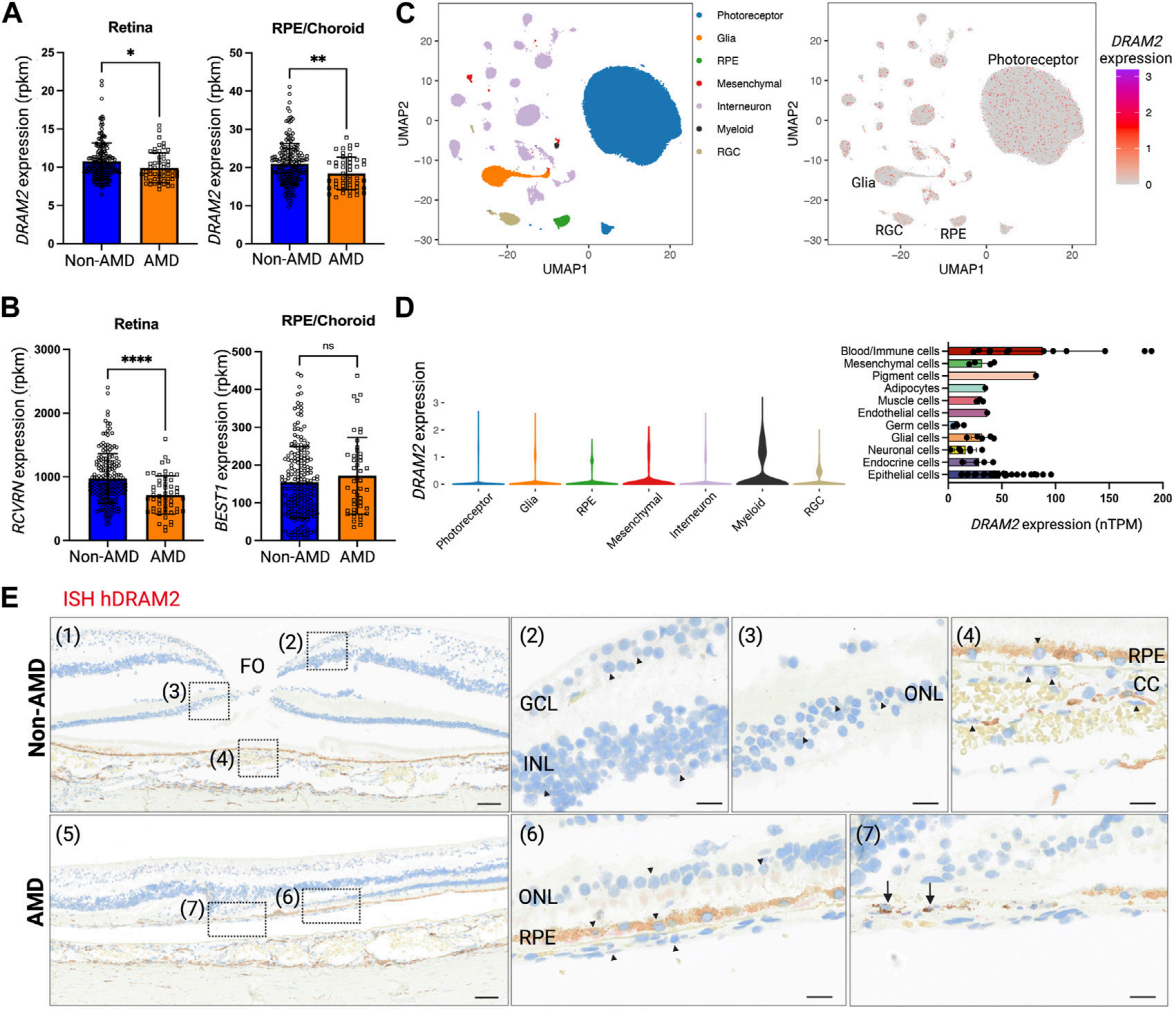
Human *DRAM2* is ubiquitously expressed in the eye. **(A)** *DRAM2* expression (rpkm) in human non-Age-related Macular Degeneration (AMD) or AMD retina and retinal pigment epithelium (RPE)/choroid samples (*n* = 49 to 198, Unpaired *t*-test, *p < 0.05, **p < 0.01). **(B)** Expression of key markers for photoreceptors (*RCVRN*) and RPE cells (*BEST1*) in human non-AMD or AMD retina and RPE/choroid samples (*n* = 49 to 198, unpaired *t*-test, ****p < 0.0001, ns: not significant *p* > 0.05). **(C)** *DRAM2* expression in single nuclei RNA sequencing of human non-AMD eyes (*n* = 4); data from (Orozco et al., 2020). **(D)** *DRAM2* expression in different cell types (left panel, snRNA seq from non-AMD eyes (*n* = 4); data from (Orozco et al., 2020)) and other organs (right panel, scRNA seq data compiled from the Human Protein Atlas (Karlsson et al., 2021)). **(E)** *In situ* hybridization (ISH) of *DRAM2* mRNA in human non-AMD (top panels) and AMD (bottom panels) donor eyes. Representative images from *n* = 4 non-AMD eyes and 8 AMD eyes. Nuclear stain is in blue, natural pigment in the RPE is in brown and the ISH signal in red. (1) Area of fovea (FO). (2) *DRAM2* signal is sparsely disseminated in the ganglion cell layer (GCL) as well as inner nuclear layer (INL). (3) *DRAM2* signal is also sparsely disseminated in the outer nuclear layer (ONL), i.e., associated with photoreceptors nuclei. (4) Sparse *DRAM2* signal localizes to cells of the choriocapillaris (CC) and retinal pigment epithelium (RPE). (5) Lesion edge in an AMD donor eye. (6) Adjacent to the lesion, sparse *DRAM2* signal is disseminated in the stretch of contiguous RPE and the few residual photoreceptors in the ONL. (7) Within the lesion, a relatively abundant DRAM2 signal is associated with cells in the area of the destroyed RPE (black arrows). (FO, fovea; GCL, ganglion cell layer; INL, inner nuclear layer; ONL, outer nuclear layer; RPE, retinal pigment epithelium; CC, choriocapillaris). Scale bar: 100 µm on panels (1) and (5), and 20 µm on panels (2), (3), (4), (6) and (7).

Rare biallelic *DRAM2* variants causing putative loss of DRAM2 function have been associated with retinal dystrophy (El-Asrag et al., 2015; Sergouniotis et al., 2015; Birtel et al., 2018; Kuniyoshi et al., 2020; Krašovec et al., 2022). Early in the third decade of life, patients become symptomatic, suffering from maculopathy and progressive central visual loss. To know if DRAM2 could also be involved in more common maculopathies, such as Age-related Macular Degeneration (AMD), we investigated whether *DRAM2* expression level in the eye was altered in AMD patients. Most AMD patients have early or intermediate AMD, characterized by build-up of drusen under the retina, and mild to no visual symptoms. However, they become at risk of severe vision loss as the disease progresses to advanced AMD, characterized by either degeneration of macular photoreceptors and their underlying retinal pigment epithelium (RPE) and/or growth of pathogenic blood vessels from the choroid into the retina. Using bulk RNA sequencing of macula and non-macula retinas and RPE/Choroids from human donor eyes (99 donors had no history of ocular disease and 23 donors were diagnosed with advanced AMD (Orozco et al., 2020)), we found that *DRAM2* expression was slightly lower in AMD retinas and RPE/Choroids compared to non-AMD controls *(p* < 0.05 and *p* < 0.01, respectively; Figure 1A). *DRAM2* has been described as expressed in photoreceptors and RPE cells in mice (El-Asrag et al., 2015), therefore, we checked if decreased *DRAM2* expression in AMD samples could simply reflect degeneration and loss of these particular cells during the disease process. *RCVRN*, a photoreceptor marker, showed decreased expression in AMD retinas compared to non-AMD retinas (*p* < 0.0001), confirming substantial photoreceptor loss (Figure 1B, left). However, *BEST1*, a RPE cell marker, had similar expression levels in AMD and non-AMD RPE/Choroid samples (Figure 1B, right), suggesting that RPE atrophy was minimal in these patient samples. When assessing association of DRAM2 expression to either RCVRN or BEST1 expression within each sample, we did not find significant correlation, suggesting that lower DRAM2 expression in AMD samples is not just due to photoreceptor or RPE cell loss (retina *R* ^2^ = 0.28, and RPE/Choroid *R* ^2^ = 0.0001).

To further investigate *DRAM2* expression at the cell type level, we leveraged single-nucleus RNA sequencing (scNucSeq) data from 4 donor eyes with no retinal disease diagnosis (Orozco et al., 2020). We found that *DRAM2* is ubiquitously expressed in the human eye, with expression detected notably in neurons (photoreceptors, interneurons and retinal ganglion cells (RGCs)), RPE cells, glia, mesenchymal and myeloid cells (Figures 1C, D left). This absence of cell type-specific *DRAM2* expression uncovered in the eye was also confirmed in other organs using single cell transcriptomics datasets from 13 tissues and blood, where *DRAM2* was ubiquitously expressed in all the different cell types identified (the Human Protein Atlas (Karlsson et al., 2021)) (Figure 1D, right).

Finally, we confirmed broad *DRAM2* expression in the human and mouse eyes by *in situ* hybridization (ISH) (Figure 1E and Supplementary Figure S1A). In non-AMD donor eyes, *DRAM2* mRNA was sparsely detected in the ganglion cell layer (GCL), the inner nuclear layer (INL), the outer nuclear layer (ONL), the retinal pigment epithelium (RPE) and the choriocapillaris (CC) (Figure 1E, top panels: representative image in the macula). In advanced dry AMD donor eyes, *DRAM2* mRNA was also sparsely detected in all the different retinal layers (Figure 1E, bottom panels, representative image in the macula). Of note, a relatively abundant signal was associated with a very few cells in the choriocapillaris, where the RPE was destroyed in the AMD lesions (Figure 1E, arrows in panel (7)). Unfortunately, no anti-DRAM2 antibody with a satisfactory specificity profile was identified to be able to confirm these finding at the protein level.

In conclusion, we found that *DRAM2* is ubiquitously expressed in the human eye and expression levels are low and comparable across the different retinal layers. No cell type-specific expression was observed in the eye or other tissues for which data were available. We observed expression changes at the transcriptional level when investigating AMD eyes, with overall lower expression in diseased retinas and RPE/choroids.

### 3.2 DRAM2 loss in human pluripotent stem cell (hPSC)-retinal organoids results in the presence of extra cells with a mesenchymal gene signature

**FIGURE 2:**
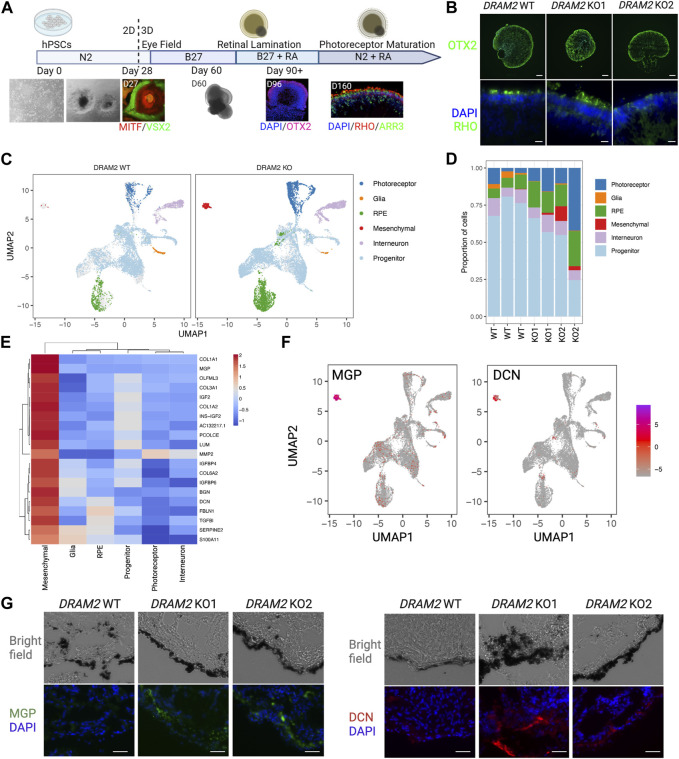
Absence of transcriptional changes in hPSC-retinal organoids knockout for *DRAM2* but presence of an extra cell cluster with mesenchymal signature **(A)** Overview of the differentiation protocol of human pluripotent stem cells (hPSC)-derived retinal organoids. MITF: RPE progenitor marker; VSX2, neuronal progenitor marker; OTX2, retinal progenitor marker; RHO, rod photoreceptor marker; ARR3, cone photoreceptor marker. **(B)** Representative immunofluorescent images of *DRAM2* WT, KO1, and KO2 hPSC-derived retinal organoids. Scale bars in upper panels: 100 µm. At day 160, retinal organoids express retinal progenitor (OTX2) and photoreceptor markers (RHO). Scale bars in bottom panels: 20 µm. **(C)** UMAP representation of single cell RNA sequencing (scRNAseq) of *DRAM2* WT and KO hPSC-derived retinal organoids identifying 6 clusters of different cell types. **(D)** Proportion of the different cell types from identified cell clusters from the scRNAseq of *DRAM2* WT and KO retinal organoids. **(E)** Heatmap showing expression of the mesenchymal markers by cell type (pseudobulk, genotypes pooled). **(F)** UMAP representation of *Matrix Gla Protein* (*MGP*) and *decorin* (*DCN*) expression in the scRNAseq of retinal organoids, showing predominant expression in the mesenchymal cluster. **(G)** Representative images of immunolabeling of MGP (left) and DCN (right) in *DRAM2* WT, KO1, and KO2 hPSC-derived retinal organoids. Scale bars: 25 µm.

Since *DRAM2* is ubiquitously expressed in the human retina, we investigated the consequences of *DRAM2* loss in human retinal organoids, as they contain most retinal cell types (Cowan et al., 2020; Sridhar et al., 2020). First, we created two independent hPSC *DRAM2* biallelic knockout lines (KO1 and KO2) using CRISPR/Cas9 (Supplementary Figure S1E, F). Then, we generated retinal organoids by directed differentiation of hPSC wild-type (WT), *DRAM2* KO1 and KO2, as previously described (Figure 2A; [43–46]. Eye cups expressing early markers for the RPE (MITF) and neural retina (VSX2) were detected after 1 month of differentiation (Figure 2A). These eye cups were manually detached and cultured to form three-dimensional retinal organoids with photoreceptor progenitor cells (OTX2) at 2 months, and rod and cone photoreceptor cells (RHO positive and ARR3 positive cells, respectively) at 5 months (Figure 2A). *DRAM2* WT, KO1 and KO2 hPSC-retinal organoids showed some variability in shape and size that is inherent to organoid directed differentiation; however, no obvious consistent morphological differences were observed between the two genotypes. They all showed spontaneous retinal lamination with photoreceptors localized on the outer layer in contact with the culture media (Figure 2B). To further analyze the hPSC-retinal organoids, we matured them until 12 months of age and performed scRNA sequencing. Clustering the cells based on their gene expression identified five distinct cell populations in both the *DRAM2* WT and KO retinal organoids including: photoreceptors, glial cells, RPE cells, interneurons, and progenitor cells (Figure 2C). Cell composition was variable between the different retinal organoids and our limited number of replicates (*n* = 3 for *DRAM2* WT and *n* = 4 for *DRAM2* KO) does not allow us to determine if any of the cell type proportion differences between WT and KO organoids are biologically relevant (Figure 2D). Differentially Expressed Genes (DEGs) analysis of the whole retinal organoids did not show any significant differences between *DRAM2 WT* and *KO* organoids, with the exception of *PRSS56* (FDR = 0.048) (Supplementary Figure S1B). This gene has been described as being expressed mainly first in retinal progenitors and then Muller Glia in the mouse retina (Paylakhi et al., 2018). Since these are cell types that are overall more present in *DRAM2 WT* organoids, the lower *PRSS56* expression in *DRAM2 KO* organoids is most likely reflecting the presence of a lower cell number expressing it (Supplementary Figure S1B). Indeed, when pseudobulk DEG analysis was done comparing *DRAM2 WT* and *KO* within each cell type, no genes were identified as significantly up or downregulated.

We were able to notice that an extra cluster was present in the *DRAM2* KO retinal organoids (average cell identified: 1 cell per WT organoid and 67 cells per KO organoid) (Figure 2D). We identified this extra *DRAM2* KO cluster as mesenchymal cells based on cluster marker genes (Figure 2E). We validated the existence of the extra cluster in *DRAM2* KO organoids using Matrix Gla Protein (*MGP*) and Decorin (*DCN*) as marker genes (Figure 2F). Using immunolabeling, we confirmed that they were both not detected in *DRAM2* WT organoids. Interestingly, we found that MGP and DCN were expressed by cells localized next to RPE cells in the *DRAM2* KO organoids, and not within the self-laminated neuroretina of the hPSC-organoids (Figure 2G).

In summary, we found that *DRAM2* loss in hPSC-retinal organoids does not affect cells at the transcriptional level, but induces the presence of additional mesenchymal cells, which localize near RPE cells.

### 3.3 DRAM2 loss exacerbates toxicity-induced human RPE cell death *in vitro*

**FIGURE 3:**
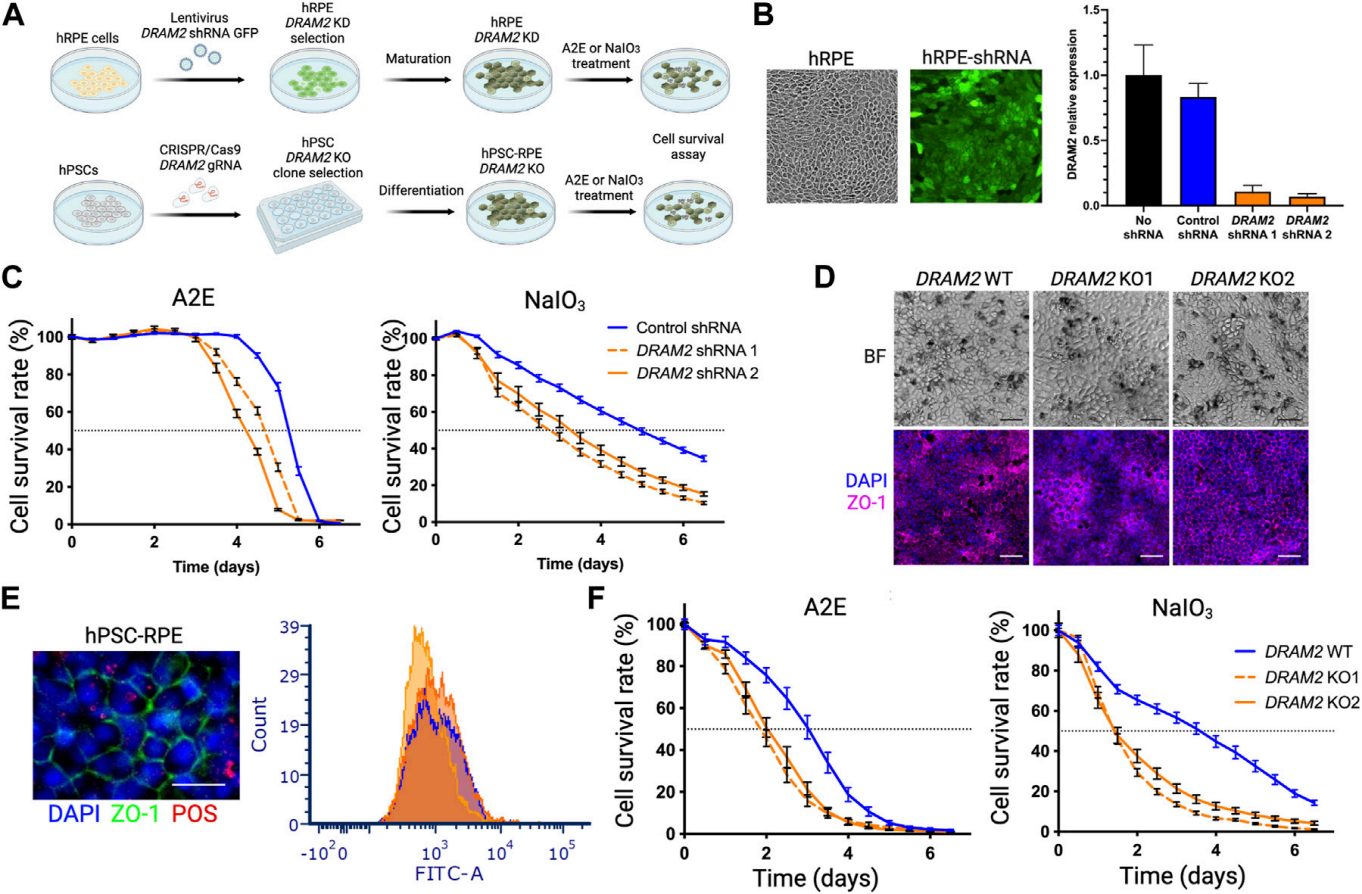
*Dram2* loss exacerbates toxicity-induced human RPE cell death *in vitro* **(A)** Experimental design of loss of *DRAM2* experiments in human cells. hRPE, human primary retinal pigment epithelial (RPE) cells; KD, knockdown; A2E, N-retinylidene-N-retinylethanolamine; NaOI_3_, Sodium Iodate; hPSC, human pluripotent stem cells; KO, Knockout. **(B)** Representative image of hRPE after infection with lentivirus expressing *DRAM2* shRNA and GFP (left) and knockdown efficiency of the two different *DRAM2* shRNAs assessed by quantitative RT-PCR (qPCR) analysis of *DRAM2* expression (right) **(C)** hRPE cell survival following treatment with A2E (30 μM; left) and NaIO_3_ (5 mM; right). Dotted horizontal line marks the median survival (50% of the cells alive). **(D)** Differentiation of *DRAM2* WT and KO human pluripotent stem cells (hPSCs) into RPE (hPSC-RPE) showing pigmentation (top row, BF: Bright Field) and expression of RPE maker ZO-1 (bottom row). Scale bars: 50 µm. **(E)** Representative immunofluorescent image (left) of RPE marker ZO-1 (green) in hPSC-RPE *DRAM2* WT, KO1, and KO2 and phagocytosis of FITC-labeled Photoreceptor Outer Segments (POS; red). Scale bar: 20 µm. Phagocytosis capacity of the cells was assessed by quantification of cells with internalized FITC-POS by flow cytometry (*DRAM2* WT: blue line, *DRAM2* KO1 and 2: orange lines). **(F)** hPSC-RPE cell survival following treatment with A2E (30 μM; left) and NaIO_3_ (5 mM; right). Dotted horizontal line marks the median survival.

Since we observed the strongest *DRAM2* mRNA signal in cells localized within the RPE layer in AMD lesions (Figure 1E) and identified extra mesenchymal cells next to RPE cells in *DRAM2* KO retinal organoids (Figure 2G), we investigated the consequences of *DRAM2* loss in human RPE cells. We used two different *in vitro* systems: 1) lentivirus-shRNAs to knockdown *DRAM2* expression in human primary RPE cells (hRPE) and 2) CRISPR/Cas9 to knockout *DRAM2* in human pluripotent stem cell-derived RPE cells (hPSC-RPE) (Figure 3A). RPE cells with *DRAM2* knockdown or knockout were fully differentiated before being challenged by either *N*-retinylidene-*N*-retinylethanolamine (A2E) or sodium iodate (NaIO_3_). At the cellular level, A2E is a toxic visual cycle by-product found in lipofuscin deposits within RPE cells (Crouch et al., 2015; Parmar et al., 2018) NaIO_3_ is known to induce oxidative stress, complement cascade activation, necroptosis and apoptosis (Balmer et al., 2015; Hanus et al., 2016; Berkowitz et al., 2017; Enzbrenner et al., 2021).

We first induced *DRAM2* knockdown in primary hRPE cells. High lentiviral infection efficiency was validated using GFP co-expression with the shRNAs, and two independent shRNAs targeting *DRAM2* were used with an approximately 10-fold knockdown efficiency determined by qRT-PCR (Figure 3B). The hRPE cells were then allowed to fully mature for at least 4 weeks before A2E or NaIO_3_ treatment and cell survival analysis. *DRAM2* knockdown in hRPE exacerbated both A2E- and NaIO_3_-induced cell death as compared to control RPE cells (Figure 3C). After A2E treatment, 50% of the hRPE cells expressing *DRAM2* shRNA1 or shRNA2 died after 114 and 102 h respectively, whereas the median survival for the hRPE cells expressing the control shRNA was 126 h. Similarly, after NaIO_3_ treatment, 50% of the hRPE cells expressing *DRAM2* shRNA1 or shRNA2 died after 64 and 78 h respectively, whereas the median survival for the hRPE cells expressing the control shRNA was 118 h (Figure 3C).

We then replicated these findings in *DRAM2* knockout hPSC-RPE cells. *DRAM2* WT, KO1 and KO2 hPSC were differentiated into RPE cells and allowed to mature for at least 4 weeks (Figure 3D). No obvious phenotypic differences were observed between hPSC-RPE wild-type (WT) and KO1 or KO2 during the directed differentiation process, showing that DRAM2 does not play a critical role in RPE differentiation and survival. All hPSC-RPE cell lines formed a monolayer, became pigmented, and expressed the RPE marker ZO-1 (Figure 3D). Furthermore, mature hPSC-RPE cells were similarly functional as both *DRAM2* KO1 and KO2 cells were able to phagocytize photoreceptor outer segments (POS) as efficiently as the WT cells (Figure 3E). However, after A2E treatment, 50% of the hPSC-RPE KO1 and KO2 died after 42 and 50 h respectively, whereas the median survival for the hPSC-RPE WT was 72 h. Similarly, after NaIO_3_ treatment, 50% of both the hPSC-RPE KO1 and KO2 died after 34 h, whereas the median survival for the hPSC-RPE WT was 86 h (Figure 3F).

In conclusion, *DRAM2* loss by either knockdown or knockout in human RPE cells resulted in decreased survival after challenges by A2E or NaIO_3_ *in vitro*, showing that DRAM2 plays a role in resistance of human RPE cells to stress-induced cell death.

### 3.4 Dram2 loss causes mild age-related retinal dystrophy with absence of functional deficit in mice

**FIGURE 4:**
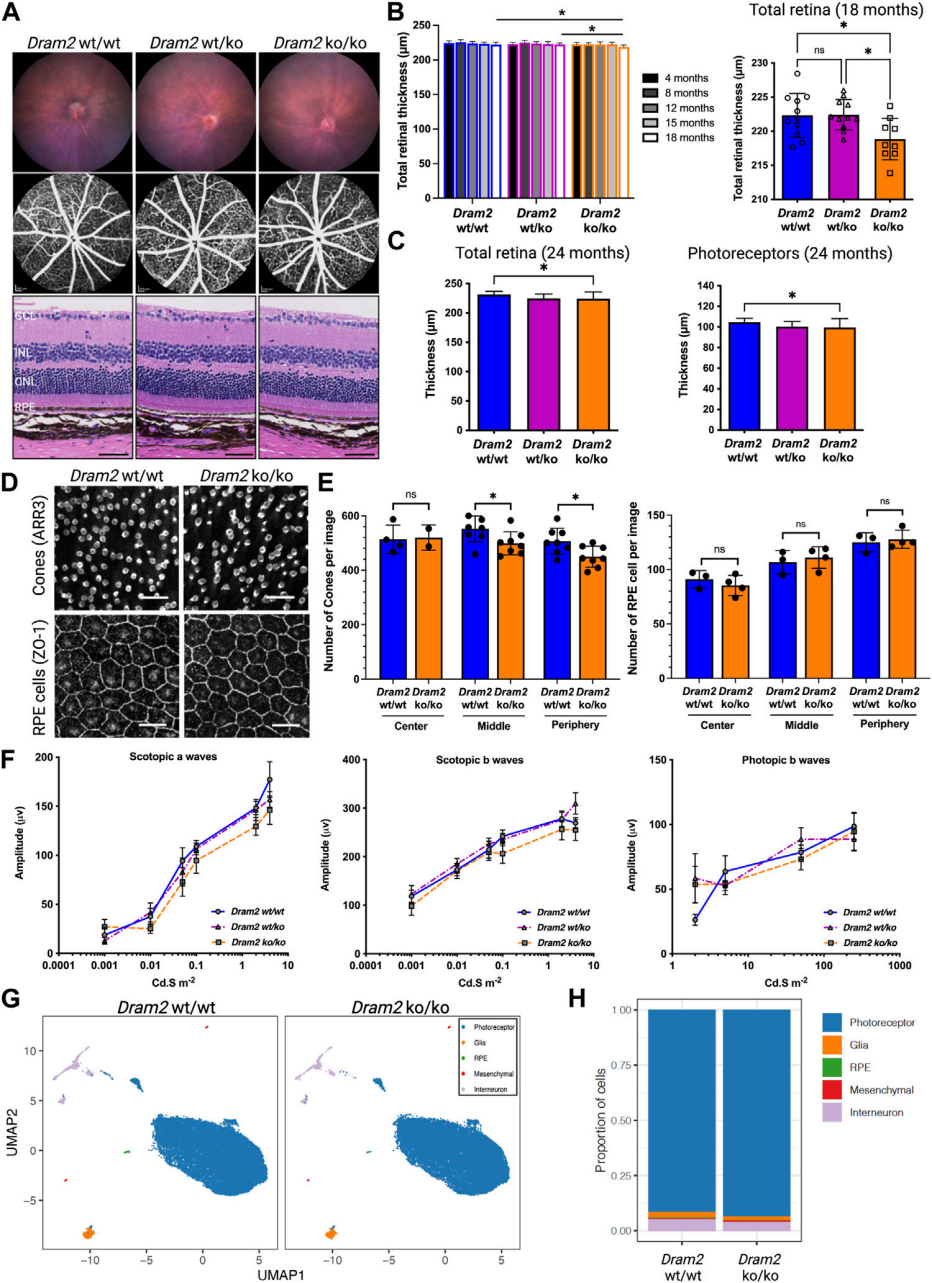
*Dram2* loss causes very mild age-related photoreceptor degeneration with absence of functional deficit or transcriptional changes in mice. **(A)** Fundus imaging (top row) and fundus angiography (middle row) at 4-month of age. Histopathological (bottom row) analyses of *Dram2* wt/wt, wt/ko, and ko/ko mice at 24 months (Hematoxylin Eosin, 200 µm from the optic nerve head). GCL, ganglion cell layer; INL, inner nuclear layer; ONL, outer nuclear layer; RPE, retinal pigment epithelium. Scale bar, 50 µm. **(B)** Optical coherence tomography (OCT) analysis followed by automated segmentation of retinal thickness in *Dram2* wt/wt, wt/ko, and ko/ko. Time course of retinal thickness of all genotypes from 4 to 18-month of age (left; *n* = 8–14 mice per genotype; one-way ANOVA *p < 0.05) and between the genotypes at 18-month of age (right; *n* = 9–11 mice per genotype; one-way ANOVA *p < 0.05; ns, not significant). **(C)** Total retinal thickness at 24 months (left) and photoreceptor layer thickness (right) of *Dram2* wt/wt, wt/ko, and ko/ko mice (*n* = 16–27 mice per genotype, one-way ANOVA *p < 0.05). Automated 8-layer retinal segmentation was performed on this cohort of mice and only the photoreceptor layer (Outer nuclear layer (ONL) + inner segment + outer segment) showed significant difference between *Dram2* wt/wt and *Dram2* ko/ko mice (other layers shown in Supplementary Figure S3A). **(D)** Representative immunofluorescent image of cone photoreceptor cells (ARR3; top row) and RPE cells (ZO-1; bottom row) from peripheral retina. Scale bar, 25 µm. **(E)** Quantification of cone photoreceptor cells (left) and RPE cells (right) in *Dram2* wt/wt and *Dram2* ko/ko retinas (Mann-Whitney test, *p < 0.05, ns, not significant). **(F)** Electroretinography (ERG) analysis of *Dram2* wt/wt, wt/ko, and ko/ko mice at 21 months. (mean±SEM, *n* = 9–10 mice per genotype). **(G)** UMAP representations of single cell RNA sequencing (scRNAseq) analysis of *Dram2* wt/wt and ko/ko retinas **(H)** Proportion of cell types per genotype identified from the scRNAseq analysis.

To further study the effect of *Dram2* loss on retinal homeostasis, a constitutive CRISPR/Cas9 knockout (ko) C57BL/6J mouse strain was generated (Supplementary Figure S1C), and the disruption was confirmed by genomic DNA sequencing. The mouse ko region of Exon 4 corresponds to known patient biallelic mutations that cause retinal dystrophy (Supplementary Figure S1D; (El-Asrag et al., 2015)). *Dram2 wt/wt*, *wt/ko* and *ko/ko* mice were aged up to 24 months and no gross phenotypic differences were observed or detected by necropsy analysis. Ocular examination by fundus imaging, fluorescence angiography, and histological staining also did not reveal obvious phenotypic abnormalities in *Dram2 wt/ko* and *ko/ko* mice (Figure 4A). However, after 18 months, the total retinal thickness measured by live spectral-domain optical coherence tomography (SD-OCT) was slightly, but significantly decreased in *Dram2 ko/ko* mice as compared with their wild-type and heterozygous littermates (5 µm loss after 18 months, *p* < 0.05; Figure 4B). To determine the cell type contributing to loss in retinal thickness, a second cohort of *Dram2 wt/wt*, *wt/ko* and *ko/ko* mice was generated and aged. A small significant total retinal thickness loss in *Dram2 ko/ko* mice was replicated (reaching 7 µm loss after 24 months, *p* < 0.05; Figure 4C). The loss was found to be due to photoreceptor degeneration, while none of the other retinal layers (retinal nerve fiber layer (RNFL), retinal ganglion cell (RGC) layer, inner nuclear layer (INL), inner plexiform layer (IPL) or the choriocapillaris) showed thinning (Figure 4C and Supplementary Figure S3A). Since human *DRAM2* mutations cause cone-rod dystrophy, we investigated if cone photoreceptors were also affected in the mouse ko. Indeed, quantification of cone arrestin-3 (ARR3) positive cells at 4 months already identified significant loss in cone photoreceptor cells in *Dram2 ko/ko* mice (*p* < 0.05; Figures 4D, E). RPE cells were also quantified at the same age and no significant difference was detected in the number of ZO-1 positive cells from the center (closest to the optic nerve head), middle, and periphery (Figure 4E). This suggests that the age-related cone loss observed in *Dram2 ko/ko* mice is not secondary to RPE cell loss. To determine if photoreceptor loss caused visual deficit in *Dram2 ko/ko* mice, electroretinography (ERG) was performed on 21-month-old mice. No differences in rod (scotopic) or cone (photopic) responses were detected between *Dram2 wt/wt*, *wt/ko* and *ko/ko* mice (Figure 4F). These results show that loss of *Dram2* leads to age-related photoreceptor degeneration, but the severity of the retinal dystrophy is not sufficient to impact visual function in mice.

To investigate ongoing gene expression differences between *Dram2 wt/wt* and *ko/ko* mice, single-cell RNA sequencing (scRNAseq) was performed in 12-month-old retinas. Unsupervised cluster analysis identified five distinct cell populations in the retinas, including photoreceptors, glia, RPE cells, mesenchymal cells and interneurons (Figure 4G). The percentage of the different cell types was not significantly different between the two genotypes with the exception of the RPE cells (0.34% ± 0.005 in *Dram2 wt/wt* retinas and 0.19% ± 0.07 in *Dram2 ko/ko* retinas). However, presence of RPE cells in the cell preparation is an artifact of retina isolation and only a total of 10–25 RPE cells per retina were identified by scRNAseq. As expected, the vast majority of cells were photoreceptors (91.4% ± 4.4 in *Dram2 wt/wt* retinas and 93.7% ± 2.2 in *Dram2 ko/ko* retinas, Figure 4H). Significant Differentially Expressed Genes (DEGs) were identified only in interneurons (51 genes), mesenchymal cells (56 genes), and RPE cells (5 genes) (Supplementary Table S1). Strikingly, no significant DEGs were detected in photoreceptor cells from *Dram2 wt/wt* and *ko/ko* retinas (FDR <0.05).

In conclusion, although no transcriptional changes were detected at 12 months, *Dram2* loss causes a mild age-related retinal degeneration starting at 18 months, which is restricted to photoreceptor cells and not severe enough to affect vision in mice even at 21 months.

### 3.5 Dram2 loss exacerbates choroidal neovascular lesions but not retinal degeneration caused by acute photoreceptor or RPE injury

**FIGURE 5:**
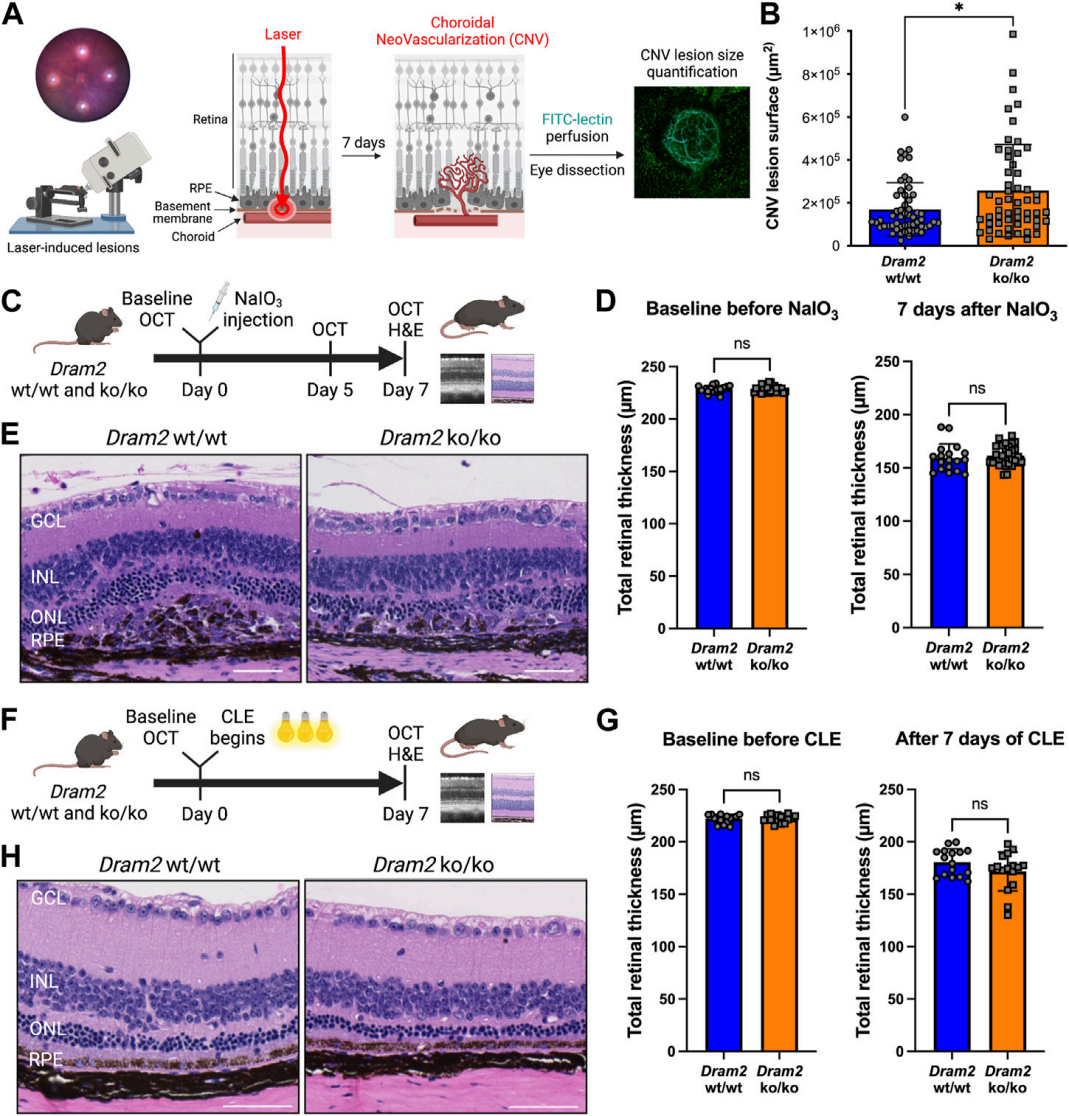
*Dram2* loss exacerbates choroidal neovascularization lesions but does not exacerbate retinal dystrophy in mouse pre-clinical models involving photoreceptor or RPE injury **(A)** Experimental design of the laser-induced choroidal neovascularization (CNV) mouse pre-clinical model. **(B)** Quantification of CNV lesion surface size (in µm^2^) 7 days after disruption of the basement membrane by the laser burn in *Dram2* wt/wt and ko/ko retinas (*n* = 55 lesions per genotype, unpaired *t*-test, *: *p* < 0.05). **(C)** Experimental design of the sodium iodate (NaIO_3_) degeneration model (three independent cohorts, *n* = 9 to 15 mice per genotype at 5–18 weeks of age). OCT, optical coherence tomography; H&E, hematoxylin and eosin. **(D)** Retinal thickness in the NaIO3 experiment measured by OCT in *Dram2* wt/wt and ko/ko mice at baseline (left) and 7 days (right) after NaIO_3_ treatment. Unpaired *t*-test; ns, not significant. **(E)** Histopathological analysis of *Dram2* wt/wt (left) and ko/ko (right) retinas after NaIO_3_ treatment. **(F)** Experimental design of the constant light exposure (CLE) degeneration model (three independent cohorts, *n* = 8 mice per genotype at 33–40 weeks of age). **(G)** Retinal thickness in the CLE experiment measured by OCT in *Dram2* wt/wt and ko/ko mice at baseline (day 0, left) and after 7 days of exposure (right). Unpaired *t*-test; ns, not significant. **(H)** Histopathological analysis of *Dram2* wt/wt and ko/ko retinas after CLE (day 7). Scale bars = 50 µm. GCL, ganglion cell layer; INL, inner nuclear layer; ONL, outer nuclear layer; RPE, retinal pigment epithelium.

Since *Dram2* loss causes a slow-progressing and mild retinal dystrophy in mice, we tested if the phenotype could be exacerbated by additional environmental stress. We selected three pre-clinical models commonly used to model AMD: 1) the laser-induced choroidal neovascularization (CNV) model (Lambert et al., 2013), 2) the sodium iodate model (NaIO_3_), in which photoreceptor degeneration happens secondarily to RPE-specific toxicity-induced cell death (Balmer et al., 2015; Zhang et al., 2021) and 3) the constant light exposure model (CLE), in which phototoxicity directly causes photoreceptor death (Natoli et al., 2016), (Figure 5).

Because we identified an extra mesenchymal-like cluster in *DRAM2* KO organoids (Figure 2C) and because *DRAM2* silencing has been associated with increased tumor growth and resistance to apoptosis (Park et al., 2009; Bai et al., 2016; Wudu et al., 2019), we wanted to know if *DRAM2* loss could have consequences on cell survival, proliferation or/and migration of mesenchymal-like cells. Since we identified *DRAM2* expression changes in the choriocapillaris in AMD eyes (Figure 1E), we used the laser-induced choroidal neovascularization (CNV) pre-clinical model to challenge cells around this area (Figure 5A). In this model, the Bruch’s membrane between the choriocapillaris and the RPE is disrupted using a laser burn, and the resulting CNV lesions involve endothelial cells, pericytes, fibroblasts and mesenchymal cells, with blood vessels growing into the retina (Lambert et al., 2013). One week after the laser induction, the CNV lesion surface areas are quantified. Interestingly, a significant increase in the lesion size in *Dram2 ko/ko* mice was detected, with mean lesion size being 256,857 μm^2^, compared to the *Dram2 wt/wt* mean lesion size of 169,340 μm^2^ (Figure 5B, *p*-value = 0.0102).

To further investigate the role of Dram2 loss at the cellular level, we isolated choroidal and RPE cells from *Dram2 wt/wt* and *ko/ko* mice. Eyes were enucleated and after removal of the anterior chamber, the lens and the retina, the choroid containing RPE cells was dissected, dissociated and cultured for 7 days (Supplementary Figure S2A). The cells were collected and scRNAseq was performed. Clustering cells based on their gene expression identified 8 clusters of different cell types including: RPE cells, two distinct fibroblast clusters (fibroblast 1 and 2), fibroblast proliferating cells, fibroblast-like cells, endothelial cells, pericytes, and myeloid cells (Supplementary Figure S2B). There were no significant differences in cell type composition between *Dram2 wt/wt* and *ko/ko* RPE/Choroids (Supplementary Figure S2C) and most of the cells were fibroblastic cells expressing characteristic cell markers (Supplementary Figure S2D). The fibroblasts (1 and 2), fibroblasts proliferating, and fibroblast-like cells were sub-clustered further, but again, no differences were revealed between the *Dram2 wt/wt* and *ko/ko* samples (Supplementary Figure S2E). Interestingly, when we plated the cells, we observed that the RPE/Choroid cells from *Dram2 ko/ko* mice reached 20% confluency in a little over 3 days (78 h), whereas it took a little over 5 days (126 h) for the cells from *Dram2 wt/wt* mice to reach a similar level of confluency (Supplementary Figure S2F). This finding suggests that the CNV lesion exacerbation observed in *Dram2 ko/ko* mice is caused by a choroidal cell proliferative advantage.

We then tested if the exacerbation of NaIO_3_-induced RPE cell death observed *in vitro* in absence of DRAM2 could be replicated *in vivo* (Figure 5C). Intravenous administration of NaIO_3_ induces rapid and specific RPE cell death and as the RPE plays a critical role in the maintenance and survival of the overlying photoreceptors (Boulton and Dayhaw-Barker, 2001), the RPE loss is followed by photoreceptor degeneration. SD-OCT analysis five and 7 days after treatment with NaIO_3_ revealed no significant differences in retinal thickness between *Dram2 wt/wt* and *ko/ko* mice (Supplementary Figure S3C and Figure 5D). Histological examination at day 7 confirmed thinning of the photoreceptor layer (ONL and segments) (Figure 5E), destruction of the RPE monolayer and ongoing wound healing in the subretinal space (Supplementary Figure S3B). Lesion scoring of the combined RPE and ONL damage showed similar severity in *Dram2 wt/wt* and *ko/ko* mouse retinas (Supplementary Figure S3D). These results indicate that loss of *Dram2* does not exacerbate photoreceptor loss following NaIO_3_-induced RPE damage.

Finally, we tested if the age-related photoreceptor loss observed in the *Dram2 ko/ko* mice could be exacerbated by light toxicity (Figure 5F). Mice were subjected to constant light exposure (CLE) for 7 days. SD-OCT analysis showed that after a week of exposure to bright light, *Dram2 wt/wt* mice lost 66 ± 23 µm of retinal thickness and *ko/ko* mice lost 69 ± 20 µm, revealing no significant difference between the two genotypes (Figure 5G). Histological examination confirmed that the retinal degeneration affected photoreceptors, with significant thinning of the ONL and photoreceptor inner/outer segments (Figure 5H and Supplementary Figure S3E). Lesion scoring of the damaged areas showed similar severity of retinal dystrophy in *Dram2 wt/wt* and *ko/ko* mouse retinas (Supplementary Figure S3F). These results show that loss of *Dram2* does not exacerbate photoreceptor loss following acute light-induced damage.

Collectively, our results show that *Dram2* loss in mice does not exacerbate retinal dystrophy induced by acute photoreceptor- or RPE-injury, but it exacerbates proliferation of choroidal cells, resulting in more severe choroidal neovascular lesions.

### 3.6 Integration of data from different *in vitro* and *in vivo* systems reveals complexity of human disease pathophysiology

**FIGURE 6:**
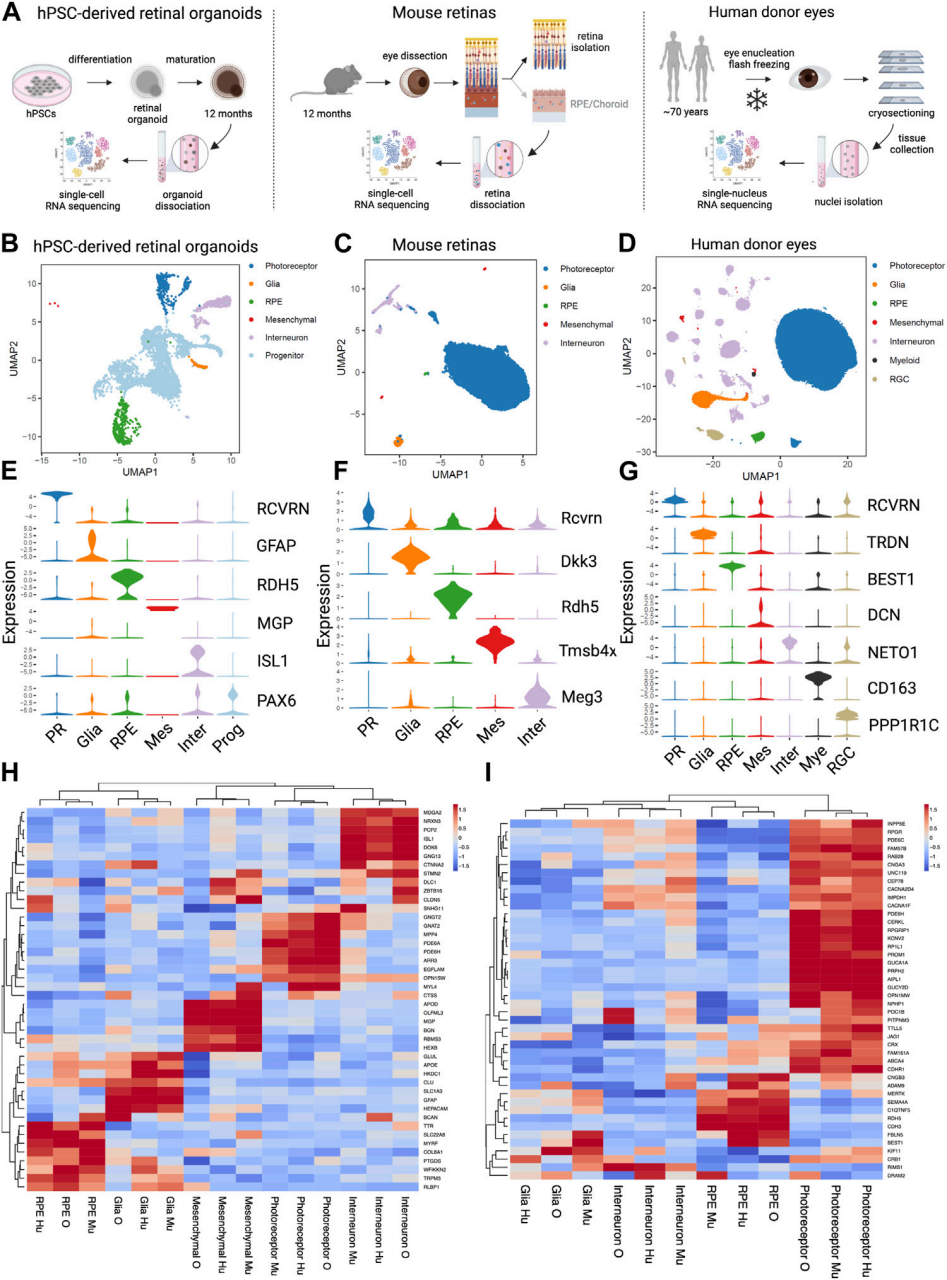
Comparative analysis of single cell analysis of human stem cell-derived retinal organoids, mouse retina and human retina **(A)** Diagrams of the experimental designs to generate single cell RNA sequencing (scRNA seq) data from human Pluripotent Stem Cells (hPSCs)-derived retinal organoids (left), scRNA seq data from mouse retinas (middle) and single nucleus RNA sequencing (snRNA seq) data from human eyes (right). **(B)** UMAP representation of the hPSC- retinal organoid scRNAseq analysis. **(C)** UMAP representation of the mouse retinal scRNAseq analysis. **(D)** UMAP representation of the human eyes snRNAseq analysis. **(E)** Violin plot of key marker genes expression for identified cell types in hPSC- retinal organoids. PR, photoreceptor; RPE, retinal pigment epithelium; Mes, mesenchymal; Inter, interneurons; Prog, progenitors. **(F)** Violin plot of key marker genes expression for identified cell types in mouse retinas. PR, photoreceptor; RPE, retinal pigment epithelium; Mes, mesenchymal; Inter, interneurons. **(G)** Violin plot of key marker genes expression for identified cell types in human eyes. PR, photoreceptor; RPE, retinal pigment epithelium; Mes, mesenchymal; Inter, interneurons; Mye, myeloid cells; RGC, retinal ganglion cells. **(H)** Heatmap of the unsupervised hierarchical cluster analysis of top marker genes per cell types identified in all three datasets. Mu, mouse retina scRNAseq; O, hPSC- retinal organoid scRNAseq; Hu, human snRNAseq. **(I)** Heatmap of the hierarchical cluster analysis of known cone-rod macular dystrophy genes expressed in the three datasets. Mu, mouse retina scRNAseq; O, hPSC-retinal organoid scRNAseq; Hu, human snRNAseq.

To gain further insights into relevance of hPSC-retinal organoids and mouse retinas to study and model human retinal dystrophies, we compared our scRNA seq datasets generated from *DRAM2* WT organoids and *Dram2 wt/wt* retinas to a previously published single nuclei sequencing of non-AMD human donor eyes (Orozco et al., 2020) (Figure 6A). Twelve-month old wild-type hPSC-retinal organoids had five main cell types, including photoreceptors, glia, RPE, interneurons, and progenitors (Figure 6B). Adult wild-type mouse retinas had three main cell types (photoreceptors, glia and interneurons) (Figure 6C) and adult human donor eyes had seven main classes of cells (photoreceptors, glia, RPE, mesenchymal, interneurons, myeloid, and retinal ganglion cells (RGC)) (Figure 6D). As described previously, very few mesenchymal cells were detected in wild-type retinal organoids, and few mesenchymal and RPE cells were captured in the mouse retina dataset. The main difference between the three datasets was that progenitors, which are absent in adult mouse and human retinas, constitute the majority of hPSC-retinal organoid cells; whereas photoreceptors are the main cell type detected in mouse and human retinas. Key marker genes for each cell type in the three datasets were identified (Figures 6E–G). Clustering the coinciding cell types between the three sample sets showed grouping of the same cell types together, and highly comparable expression of top cell type marker genes (Figure 6H). The fact that when using top marker genes, the different cell types clustered together across the three datasets is not surprising as they are very specialized cells with distinct characteristics and functions (e.g., light sensitivity for photoreceptors: OPN1MW, OPN1SW; recycling of visual cycle components by RPE cells: RBP1, RLBP1). What was less anticipated is that when we selected panels of genes involved in several biological processes common to all cell types, such as apoptosis, autophagy or lysosomal function, cell clustering once again grouped the different cell types together, independently of the dataset origin (Supplementary Figures S4A–C). These findings confirmed that hPSC-retinal organoids and mouse retinas are overall relevant models for studying human retinal biology. There are still limitations to these models. For example, when we selected a panel of genes involved in phagocytosis, cell clustering grouped the RPE cells from the hPSC-retinal organoids with the Glia cells from mouse and human retinas; while RPE cells from mouse and human retinas clustered together (Supplementary Figure S4D). This suggests that to study some human RPE biological processes such as phagocytosis, for example, using hPSC-derived RPE cells or mouse eyes will be more relevant than the RPE cells growing within the hPSC-retinal organoids.

Next, we clustered cell types using a panel of genes associated with retinal dystrophies. A compiled list of genes known to be involved in cone, cone-rod, and macular dystrophies was generated (Birtel et al., 2018; Gill et al., 2019). Clustering based on gene expression from the three sample sets showed the photoreceptors, interneurons, Glia and RPE cell types cluster by cell type and not by data source or species (Figure 6I). The vast majority of the retinal dystrophy genes (70% of them) have their strongest expression level in photoreceptor cells, and a smaller subset of genes (about 20%) is most highly expressed in RPE cells. Integration of the three datasets revealed that *DRAM2* has the highest expression in human interneurons, retinal organoid photoreceptors, and mouse RPE cells (Figure 6I). We used a combination of different systems and models to be able to uncover different aspects of DRAM2-retinopathy pathophysiology. Indeed, these particular system/cell types combination alone (i.e., photoreceptors in organoids or RPE in the mouse model) did not reveal any phenotype in absence of *DRAM2*.

In conclusion, different models such as hPSC-retinal organoids or mice can be leveraged to study and model human retinal dystrophies, however, cell type specific and species-specific expression of a gene of interest must be taken into account when picking the most relevant system. When a gene of interest is ubiquitously expressed or expressed in different cell types, such as *DRAM2*, then a combination of models is most likely to be useful to uncover the different pathophysiologic mechanisms underlying the disease.

## 4 Discussion

Before discussing our findings, we would like to acknowledge limitations of our study. First, transcript levels do not always directly correlate to protein expression, and our expression data are primarily based on mRNA expression levels. Unfortunately, we could not identify a commercially available anti-DRAM2 antibody with satisfactory selectivity validation in our hands to perform analysis at the protein level. Another limitation is that we used fetal human primary RPE cells and embryonic stem cell-derived RPE/retinal organoids to study a disease with an age-related component. Organoids accurately depict early retinal development (Sridhar et al., 2020); however, AMD occurs after decades of life and cannot be replicated *in vitro*. We aged hPSC-retinal organoids in culture for 12 months and our *Dram2 ko/ko* mice up to 24 months to recapitulate some aging features, but both models were kept in favorable and well-controlled conditions and do not mimic the environmental stress that patients experience over their lifetime. To study and potentially model *DRAM2*-retinopathy, we used knockdown or knockout in cells or mice, whereas patients carry point mutations in *DRAM2*. This choice was based on findings suggesting that patients with at least one loss-of-function variant present with earlier disease onset compared to patients carrying only missense or in-frame deletions (Sergouniotis et al., 2015). This was recently confirmed by a genotype-phenotype correlation analysis showing that non-null variants can result in milder disease (Krašovec et al., 2022). Finally, we used acute pre-clinical models. DRAM2-retinopathy is a slow progressive disease and chronic models would be more appropriate. Unfortunately, the lack of disease-relevant models is a common limitation in the age-related retinal degeneration field.

Despite some limitations, our study provides novel insight regarding different *in vitro* and *in vivo* models commonly used to study retinal dystrophies, including AMD. Since biallelic *DRAM2* variants cause retinal dystrophy with early macular involvement (El-Asrag et al., 2015; Sergouniotis et al., 2015; Birtel et al., 2018; Kuniyoshi et al., 2020; Krašovec et al., 2022), and we identify lower *DRAM2* expression in AMD patient eyes (Figure 1A), we decided to use *DRAM2* as a case study. By assessing the different phenotypes resulting from *DRAM2* loss in these various systems, our ultimate goal was to gain insights on *DRAM2*-retinopathy pathophysiology and understand the limitations of these different systems better. Interestingly, we found that *DRAM2* loss had different consequences depending on the species, the cell types analyzed and the model used. For example, while we did not see any phenotype exacerbation in the *DRAM2 ko/ko* mice after NaIO_3_ treatment (Figures 5C–E), we found that *DRAM2* loss exacerbated cell death after NaIO_3_ treatment in closely monitored human RPE cells *in vitro* (Figure 3). Similarly, the inherent variability of human PSC-derived retinal organoids did not allow us to identify differences in photoreceptor number between *DRAM2* WT and KO organoids even after a year of maturation (Figures 2B–D), however we were able to detect a mild spontaneous age-related photoreceptor dystrophy in *Dram2 ko/ko* mice (Figures 4B–E). Of note, the retinal organoid system, despite not being useful to study *DRAM2* loss consequences in photoreceptors, was critical in discovering its role in mesenchymal cell proliferation and ECM production (Figures 2C–G). This led us to test the choroidal neovascularization pre-clinical model and we observed exacerbation of the CNV lesions in absence of DRAM2 (Figure 5B). The fact that different systems did not always provide concordant results is not surprising. This is inherent to the differences in nature (*in vitro versus in vivo*), timeline (age-related *versus* acute), and readouts associated with each model. Our work highlights the importance of integration of data from different systems, of which pros and cons are taken into account, when studying complex human diseases.

As part of our study, we also performed a comparative single-cell transcriptomic analysis of the different systems. The results provide insights on which models to select when studying a particular gene or pathway. For example, *POC1B* is highly expressed in human and mouse photoreceptors but not highly expressed in hPSC-retinal organoid photoreceptors (Figure 6I). POC1B is critical for the photoreceptor connecting cilium and *POC1B* mutations cause cone-rod dystrophy (Beck et al., 2014). Lower *POC1B* expression in retinal organoid photoreceptors could reflect that their inner and outer segments are shorter and less fully developed compared to *in vivo* photoreceptors and they may not be the preferred system to study POC1B function. Another example is *CRB1*, which is expressed in photoreceptors from the three datasets. *CRB1* mutations cause variable severe retinal dystrophies with photoreceptor degeneration. However, *CRB1* is expressed in mouse and human glial cells at a higher level than in photoreceptors (Figure 6I). This is interesting because *CRB1*-associated retinal dystrophies also involve inflammation and vascular leaks (Bujakowska et al., 2012). Therefore, mice may be a better model to study *CRB1* mutations over hPSC-retinal organoids, as the organoids would not integrate vascular and glial-dependent pathogenic features to the photoreceptor degeneration. Finally, comparison of the different datasets can help with tool development, such as selection of cell type specific promoters for creation of cell line reporters or conditional mouse models. It can also help understanding existing tools better. For example, BEST1, also known as *VMD2*, is a gene highly expressed in human RPE cells and is considered a RPE cell marker (Marquardt et al., 1998; Petrukhin et al., 1998) (Figure 6G). However, we found that in the mouse retina, BEST1 is actually highly expressed in glial cells (Figure 6I). This can explain why transgenes placed under the control of a VMD2 promoter in transgenic mice do not show the intended RPE specific expression and are also expressed in Muller glia (Le et al., 2008; Ueki et al., 2009).

Knowing in which cell type a gene is highly expressed is important when considering which system to select for its investigation. However, the fact that a gene at the transcriptional level is more expressed in a cell type does not necessarily mean that this cell type will be driving disease pathophysiology. For example, in the human dataset, *CNGB3* is most highly expressed in RPE cells and to a lower extent in photoreceptors. However, *CNGB3* mutations cause achromatopsia 3, which is characterized by loss of color vision and photoreceptor degeneration (Kohl et al., 2005; Wiszniewski et al., 2007). *CNGB3* encodes a channel subunit located in the plasma membrane and is essential for generation of light-evoked electrical responses in cones (Kohl et al., 2000; Sundin et al., 2000). Studying its function in RPE cells only, where is it most highly expressed in the human eye, would not have explained how its loss of function causes achromatopsia. It is therefore important to keep in mind that expression in different cell types may translate into different consequences in term of disease mechanism, independently of the relative level of expression in the different cell types. DRAM2 turned out to be a great example of this, as it is ubiquitously expressed in the eye.

Leveraging results obtained with the different systems, we were able to recapitulate the pathognomonic clinical features of *DRAM2*-retinopathy. In patients with loss of function *DRAM2* mutations, the first sign of disease is central vision loss in the third decade of life, with early macular involvement and photoreceptor loss (Krašovec et al., 2022). In primates, the center of the macula, called the fovea, is responsible for this central vision and contains 99% of the total cone photoreceptors (Perry and Cowey, 1985). Mouse do not have a macula and fovea, so we were not able to determine if this structure is also the first affected in *Dram2* knockout animals. However, we noticed that indeed early on (4-month-old), cone photoreceptors were lost in *Dram2 ko/ko* mice compared to *wt/wt* littermates (Figure 4E). Cones in mice represent only 3% of the photoreceptors, with the majority of photoreceptors being rods (Jeon et al., 1998). The fact that when we noticed cone loss, the outer retinal layer (i.e., photoreceptor layer) was not overall thinner suggests that at that time, rods were not yet affected in the mouse mutant. As we aged the mice (18-month-old), we observed outer retinal layer thinning, showing late onset of widespread rod degeneration (Figure 4C). As the mouse retina is thought to be similar to the primate peripheral retina (Jeon et al., 1998), this finding mimics the late onset of peripheral vision loss described by patients in their fifth decade. In addition to this early cone involvement followed by rod degeneration (i.e., cone-rod dystrophy), another aspect of DRAM2-retinopathy was identified in our mouse model. Indeed, many patients have bone-spicule pigmentation, which is characterized by migration of cells from the RPE to perivascular sites and accumulation of ECM components around the blood vessels (Li et al., 1995). Although we did not observe spontaneous RPE disturbance in *Dram2 ko/ko* mice, we observed exacerbation of neovascular lesions with ECM deposition around the blood vessels, when the RPE basement membrane was physically disrupted in the CNV model. We also observed an extra cell cluster in *DRAM2* KO retinal organoids, which was characterized by a mesenchymal gene signature with high expression of ECM genes (Figure 2E). Finally, we were able to observed a decreased hRPE cell-resistance to death induced by stressful *in vitro* conditions in absence or lower expression of *DRAM2* (Figure 3). This models the distinctive peripheral RPE disruption phenotype observed in patients with nonsense mutations or decreased *DRAM2* expression (Krašovec et al., 2022). Although the different cell specific effects we uncovered are consistent with DRAM2-retinopathy and AMD clinical presentation, the exact cellular mechanisms are still unclear. It would be interesting to link DRAM2’s proposed role in autophagy to these phenotypes and understand why despite being expressed everywhere in the body, *DRAM2* mutations only affect the retina in patients. We have come a long way in term of identification of human genetics hits associated with disease, mapping their expression to individual cell types of interest and integration of these data to point towards putative pathogenic molecular mechanisms. We now have to keep developing retinal dystrophy models further, to be able to dissect these mechanisms in a disease-relevant manner and identify viable therapeutic approaches.

In conclusion, using different human pluripotent stem cell-derived *in vitro* systems and *in vivo* mouse pre-clinical models, we were able to uncover the complexity of DRAM2 function. We found that its loss in choroidal cells provided a proliferative advantage, whereas its loss in post-mitotic cells such as photoreceptor and RPE cells increased degeneration susceptibility. Our work highlights the importance of integration of data from different systems, of which pros and cons are taken into account, when studying complex human diseases. Indeed, we found that each system on its own provided only limited insights into DRAM2-disease mechanisms. However, integration of data from several systems allowed deeper understanding of the pathophysiology of retinal dystrophy associated with DRAM2 loss of function.

## Data Availability

The datasets presented in this study can be found in online repositories. The names of the repository/repositories and accession number(s) can be found below: https://www.ncbi.nlm.nih.gov/geo/, GSE220627.
