# Supplementary material for: Integration of human stem cell-derived in vitro systems and mouse preclinical models identifies complex pathophysiologic mechanisms in retinal dystrophy
Source: Front Cell Dev Biol. 2023 Aug 24;11:1252547. doi: 10.3389/fcell.2023.1252547 (PMC10483287; doi:10.3389/fcell.2023.1252547)
Supplement: Supplementary file 1 [file DataSheet1.PDF]

Supplementary Material

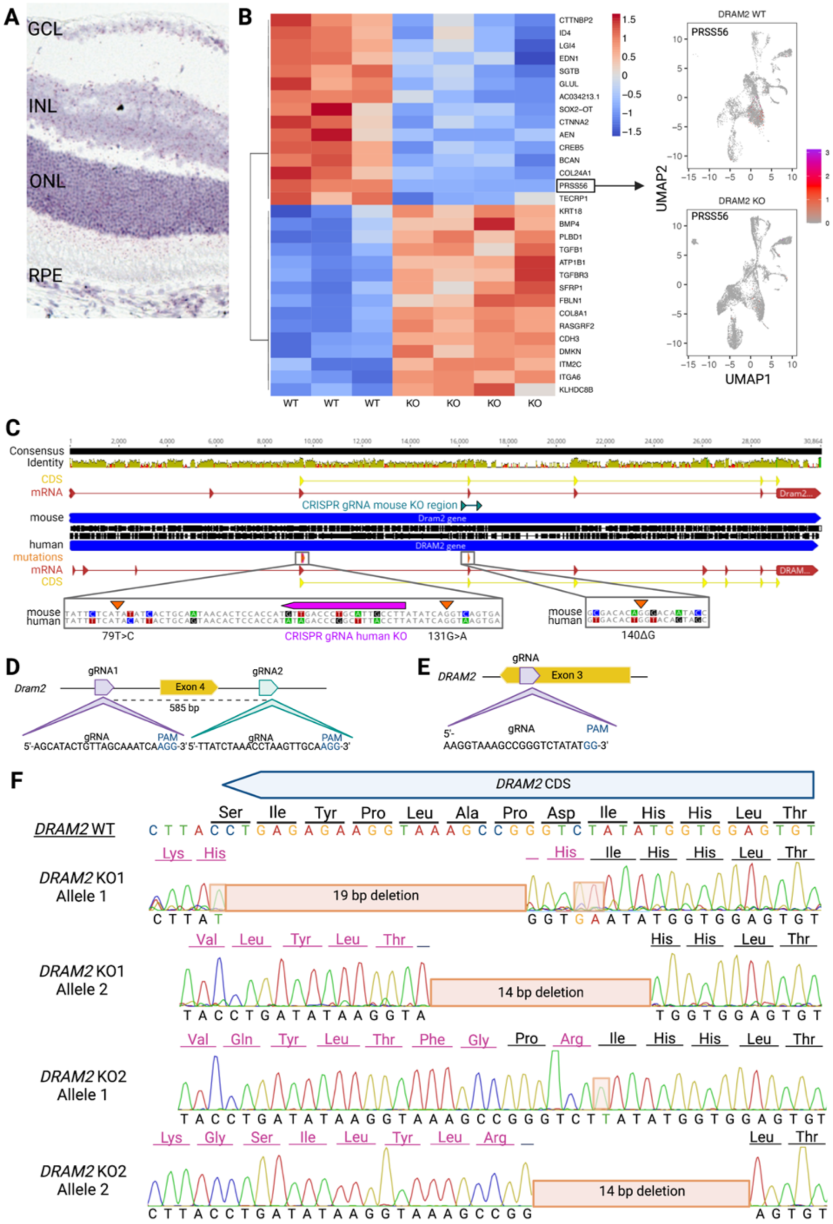

Figure S1: *Dram2* and *DRAM2* CRISPR/Cas9 knockouts (A). *In situ* hybridization of *Dram2* in wt/wt mice showing ubiquitous expression (GCL, ganglion cell layer; INL, inner nuclear layer; ONL, outer nuclear layer; RPE, retinal pigment epithelium) (B). Heatmap of the top differentially expressed genes (DEG) between *DRAM2* WT and KO retinal organoids (pseudobulk, cell types pooled). *PRSS56* was the only gene that was identified as a statistically significant DEG (FDR<0.05). UMAP of *PRSS56* expression, showing predominant expression in progenitor cells is shown on the right. (C). Alignment of mouse *Dram2* and human *DRAM2* genes, with patient specific mutations (orange arrows, El-Asrag et al., 2015), locations of CRISPR gRNA for mouse *Dram2* ko/ko (teal), and CRISPR gRNA for human pluripotent stem cell (hPSC) *DRAM2* knockout (KO) line (pink) (D). Mouse CRISPR/Cas9 ko/ko design with two gRNAs around Exon 4. (E). gRNA design for CRISPR/Cas9 knockout in Exon 3 of *DRAM2* in hPSCs. (F). Sanger sequencing of *DRAM2* KO regions in KO1 and KO2 hPSC lines. Indels show for both alleles 1 and 2 for each KO line and translation into amino acids (magenta for different amino acid acid).

Cell type	LogFC	AveExpr	PValue	FDR	ID	Gene_symbol
Interneurons	-1.725999277	5.177229704	5.25E-05	0.0210599	ENSMUSG00000113923	CT010471.2
Interneurons	-1.59033942	4.218502094	0.000165246	0.039795972	ENSMUSG00000108608	Gm6916
Interneurons	-1.524348203	3.768234684	6.50E-08	0.000278059	ENSMUSG00000064360	mt-Nd3
Interneurons	-1.522552805	3.50640662	0.000222486	0.044681186	ENSMUSG00000021741	Gm5457
Interneurons	-1.43209541	4.141086042	5.98E-05	0.022899722	ENSMUSG00000096842	Gm10736
Interneurons	-1.383432014	4.298801742	0.000215387	0.044681186	ENSMUSG00000084113	Gm14277
Interneurons	-1.355755713	4.109867409	0.000297575	0.049181607	ENSMUSG00000044071	Fam19a2
Interneurons	-1.293929624	4.340936161	0.000123572	0.03471948	ENSMUSG00000066407	Gm10263
Interneurons	-1.27993077	4.348034265	0.000289701	0.049181607	ENSMUSG00000106925	Gm20072
Interneurons	-1.178983148	4.796727424	0.000129024	0.034735655	ENSMUSG00000105913	Mir5121
Interneurons	-1.153030768	4.418478703	4.29E-05	0.019047923	ENSMUSG00000099488	Gm19774
Interneurons	-1.137766092	2.724517557	2.82E-06	0.004749383	ENSMUSG00000061762	Tac1
Interneurons	-1.108200701	4.397924858	1.35E-05	0.010739967	ENSMUSG00000037438	Uqcrh-ps1
Interneurons	-1.103301053	5.091014045	2.30E-05	0.014410403	ENSMUSG00000082456	Gm11598
Interneurons	-1.076174724	3.657980935	8.84E-06	0.00931861	ENSMUSG00000093156	Mir3076
Interneurons	-1.071894772	4.392379854	0.000282404	0.049181607	ENSMUSG00000082487	Gm11628
Interneurons	-1.0450713	3.397130263	6.80E-05	0.023867457	ENSMUSG00000030310	Slc6a1
Interneurons	-1.032689161	5.024114167	0.000233676	0.044764978	ENSMUSG00000071419	Rps15-ps2
Interneurons	-0.998921637	4.57846745	0.000123506	0.03471948	ENSMUSG00000030000	Add2
Interneurons	-0.997378022	4.518108764	0.000220725	0.044681186	ENSMUSG00000081896	Gm5389
Interneurons	-0.974705458	4.765058094	8.14E-05	0.02611514	ENSMUSG00000094955	Gm3699
Interneurons	-0.96105049	4.598140507	0.000276735	0.049181607	ENSMUSG00000106469	C030015E24Rik
Interneurons	-0.91595996	6.006219395	1.40E-05	0.010739967	ENSMUSG00000081800	Gm14200
Interneurons	-0.896254426	5.678619042	8.37E-05	0.02611514	ENSMUSG00000049517	Rps23
Interneurons	-0.88928703	5.900538712	0.000190655	0.043433284	ENSMUSG00000061808	Ttr
Interneurons	-0.888805582	8.039371628	9.90E-08	0.000278059	ENSMUSG00000060890	Arr3
Interneurons	-0.85345046	4.820499616	0.000222637	0.044681186	ENSMUSG00000075318	Scn2a
Interneurons	-0.831838618	6.031004071	6.49E-05	0.023796911	ENSMUSG00000001946	Esam
Interneurons	-0.822550685	5.440294263	0.000292742	0.049181607	ENSMUSG00000083052	Gm12285
Interneurons	-0.80973183	6.587496559	4.29E-05	0.019047923	ENSMUSG00000079942	Rpl28-ps3
Interneurons	-0.801265943	2.156474376	0.000276133	0.049181607	ENSMUSG00000023064	Sncg
Interneurons	-0.783369776	8.850633837	1.27E-05	0.010739967	ENSMUSG00000064330	Pde6h
Interneurons	-0.766800702	6.978857455	7.70E-06	0.009274122	ENSMUSG00000031558	Slit2
Interneurons	-0.696550499	7.872163229	0.000151615	0.037696987	ENSMUSG00000091449	Gm10269
Interneurons	-0.527238257	8.172526378	0.000283141	0.049181607	ENSMUSG00000017778	Cox7c
Interneurons	0.373680044	11.71011168	0.000131871	0.034735655	ENSMUSG00000004630	Pcp2
Interneurons	0.428789672	11.21835511	0.000215791	0.044681186	ENSMUSG00000001175	Calm1
Interneurons	0.657085736	7.394379296	2.74E-05	0.014410403	ENSMUSG00000005469	Prkaca
Interneurons	0.688235064	6.065829335	8.34E-05	0.02611514	ENSMUSG00000012405	Rpl15
Interneurons	0.784730477	5.674643812	2.49E-05	0.014410403	ENSMUSG00000054162	Spock3
Interneurons	0.823178194	5.447573304	0.000189382	0.043433284	ENSMUSG00000026019	Wdr12
Interneurons	0.830295093	6.564101471	1.53E-05	0.010775818	ENSMUSG00000084883	Ccdc85c
Interneurons	0.841129432	4.128348933	0.000152058	0.037696987	ENSMUSG00000028222	Calb1
Interneurons	0.843197226	6.147591873	5.08E-05	0.0210599	ENSMUSG00000036278	Macrod1

Interneurons	0.926064305	4.222068632	0.000230896	0.044764978	ENSMUSG00000073987	Ggh
Interneurons	0.959477749	6.255548967	6.00E-06	0.008425082	ENSMUSG00000035274	Tpbg
Interneurons	1.011453312	4.339006201	2.65E-05	0.014410403	ENSMUSG00000020205	Phlda1
Interneurons	1.020992264	7.103060527	6.61E-08	0.000278059	ENSMUSG00000001103	Sebox
Interneurons	1.110155932	4.221369521	0.000110063	0.03313283	ENSMUSG00000110564	Gm45697
Interneurons	1.120552751	2.900522758	3.95E-05	0.019047923	ENSMUSG00000021379	Id4
Interneurons	1.168924462	4.744656391	1.56E-06	0.003277554	ENSMUSG00000022610	Mapk12
Mesenchymal	-1.965649041	6.892619968	0.000714865	0.033072119	ENSMUSG00000022415	Syngr1
Mesenchymal	-1.837046951	7.593804643	0.000347385	0.019800928	ENSMUSG00000055541	Lair1
Mesenchymal	-1.833659986	7.354666828	0.000220877	0.013700853	ENSMUSG00000030707	Coro1a
Mesenchymal	-1.643374652	8.823983693	1.04E-07	0.000220166	ENSMUSG00000036353	P2ry12
Mesenchymal	-1.496368368	7.907391988	3.78E-05	0.006114927	ENSMUSG00000051504	Siglech
Mesenchymal	-1.471580968	8.214334434	7.51E-05	0.008340568	ENSMUSG00000036478	Btg1
Mesenchymal	-1.467736763	10.83412218	9.50E-07	0.000500834	ENSMUSG00000036896	C1qc
Mesenchymal	-1.44619643	8.150375073	0.000600155	0.030136351	ENSMUSG00000052160	Pld4
Mesenchymal	-1.423596659	11.34578675	6.88E-05	0.008055962	ENSMUSG00000038642	Ctss
Mesenchymal	-1.389942431	8.628478953	5.55E-05	0.006888148	ENSMUSG00000052336	Cx3cr1
Mesenchymal	-1.388953458	8.15639788	0.000206859	0.013309491	ENSMUSG00000040747	Cd53
Mesenchymal	-1.361465523	11.5582029	6.40E-07	0.000450214	ENSMUSG00000021665	Hexb
Mesenchymal	-1.356133198	11.08506945	5.43E-05	0.006888148	ENSMUSG00000036905	C1qb
Mesenchymal	-1.320369911	9.7421806	1.00E-05	0.00283773	ENSMUSG00000030579	Tyrobp
Mesenchymal	-1.290186233	8.509842979	8.28E-05	0.008730552	ENSMUSG00000030844	Rgs10
Mesenchymal	-1.274371847	9.63795643	1.72E-05	0.003629439	ENSMUSG00000024621	Csf1r
Mesenchymal	-1.267710786	7.878673106	0.000721345	0.033072119	ENSMUSG00000005142	Man2b1
Mesenchymal	-1.252096863	8.480728994	4.06E-05	0.006114927	ENSMUSG00000040229	Gpr34
Mesenchymal	-1.237933336	9.712716283	3.87E-05	0.006114927	ENSMUSG00000048163	Selplg
Mesenchymal	-1.228042031	9.462430616	1.08E-05	0.00283773	ENSMUSG00000023992	Trem2
Mesenchymal	-1.224392986	13.82207331	3.12E-07	0.000329345	ENSMUSG00000027447	Cst3
Mesenchymal	-1.215287426	10.42865374	1.74E-06	0.000733913	ENSMUSG00000021190	Lgmn
Mesenchymal	-1.207753674	8.502123376	9.14E-05	0.008761705	ENSMUSG00000027848	Olfml3
Mesenchymal	-1.167193685	9.021559406	0.000181882	0.012389134	ENSMUSG00000028581	Laptm5
Mesenchymal	-1.102735643	9.455442474	0.000120665	0.009787792	ENSMUSG00000058715	Fcer1g
Mesenchymal	-1.100586341	8.985121133	9.03E-05	0.008761705	ENSMUSG00000054675	Tmem119
Mesenchymal	-1.100422354	9.825810239	1.31E-05	0.003059096	ENSMUSG00000016256	Ctsz
Mesenchymal	-1.041214343	11.38324331	1.07E-05	0.00283773	ENSMUSG00000036887	C1qa
Mesenchymal	-0.980890258	11.08816732	0.000182107	0.012389134	ENSMUSG00000007891	Ctsd
Mesenchymal	-0.95271622	10.05277891	0.000153948	0.011595607	ENSMUSG00000030342	Cd9
Mesenchymal	-0.831746293	9.695850983	0.000792371	0.034814784	ENSMUSG00000021939	Ctsb
Mesenchymal	-0.677887254	11.30999497	0.001065214	0.044049739	ENSMUSG00000037706	Cd81
Mesenchymal	0.695865306	10.67337746	0.000913286	0.038694592	ENSMUSG00000031239	Itm2a
Mesenchymal	0.708323997	11.23997748	0.000545559	0.02858614	ENSMUSG00000055148	Klf2
Mesenchymal	0.755415721	10.39053085	0.001159417	0.04613606	ENSMUSG00000022010	Tsc22d1
Mesenchymal	0.774024701	13.50349168	4.52E-05	0.006361965	ENSMUSG00000023175	Bsg
Mesenchymal	0.804108661	9.56457444	0.000555729	0.02858614	ENSMUSG00000032294	Pkm
Mesenchymal	0.83965898	8.966947743	0.001088808	0.04415953	ENSMUSG00000001946	Esam
Mesenchymal	0.842181174	10.7768821	0.000396256	0.021428313	ENSMUSG00000079018	Ly6c1

Mesenchymal	0.847484087	9.233701151	0.000628903	0.030845495	ENSMUSG00000002504	Slc9a3r2
Mesenchymal	0.847588717	10.68312048	0.000344508	0.019800928	ENSMUSG00000075602	Ly6a
Mesenchymal	0.943071294	9.029678857	0.000109866	0.009613226	ENSMUSG00000009687	Fxyd5
Mesenchymal	0.946882532	8.155226979	0.001216535	0.046560906	ENSMUSG00000038729	Akap2
Mesenchymal	0.970555988	8.740882568	0.000371401	0.02061276	ENSMUSG00000002058	Unc119
Mesenchymal	0.97995715	8.287912567	0.000684208	0.032795322	ENSMUSG00000039167	Adgrl4
Mesenchymal	0.992250551	8.444830274	0.000285592	0.017208958	ENSMUSG00000038415	Foxq1
Mesenchymal	1.013969645	8.462262222	0.000160543	0.011675343	ENSMUSG00000025902	Sox17
Mesenchymal	1.018792129	7.321538253	0.001236326	0.046560906	ENSMUSG00000032292	Nr2e3
Mesenchymal	1.069925611	7.516954259	0.000758759	0.03404731	ENSMUSG00000031775	Pllp
Mesenchymal	1.118235724	7.723285398	0.001227543	0.046560906	ENSMUSG00000030247	Kcnj8
Mesenchymal	1.187331403	7.057403601	0.000917368	0.038694592	ENSMUSG00000074364	Ehd2
Mesenchymal	1.192626854	8.158058349	0.000113955	0.009613226	ENSMUSG00000030235	Slco1c1
Mesenchymal	1.212871675	7.742287018	0.000208257	0.013309491	ENSMUSG00000089945	Pakap
Mesenchymal	1.228619932	7.994756306	0.000143823	0.01123416	ENSMUSG00000020486	Sept4
Mesenchymal	1.489517784	7.228572248	3.08E-05	0.00591375	ENSMUSG00000070637	Gm694
Mesenchymal	1.877269786	7.270521537	0.000112993	0.009613226	ENSMUSG00000074896	Ifit3
RPE cells	-0.948364884	10.29737536	0.00044386	0.033644581	ENSMUSG00000029663	Gngt1
RPE cells	-0.894676613	10.44558653	0.000307148	0.033644581	ENSMUSG00000029064	Gnb1
RPE cells	0.243439667	15.96451069	0.0003863	0.033644581	ENSMUSG00000015090	Ptgds
RPE cells	1.07387703	10.50155983	0.000134588	0.025504351	ENSMUSG00000022129	Dct
RPE cells	1.484503972	10.60083248	9.52E-11	3.61E-08	ENSMUSG00000025488	Cox8b

Table S1. Significant Differentially Expressed Genes (DEGs) identified in interneurons, mesenchymal cells, and RPE cells of Dram2 wt/wt and ko/ko mouse retinas. logFC is log2(ko/ko / wt/wt), positive means higher in ko/ko and negative higher in wt/wt.

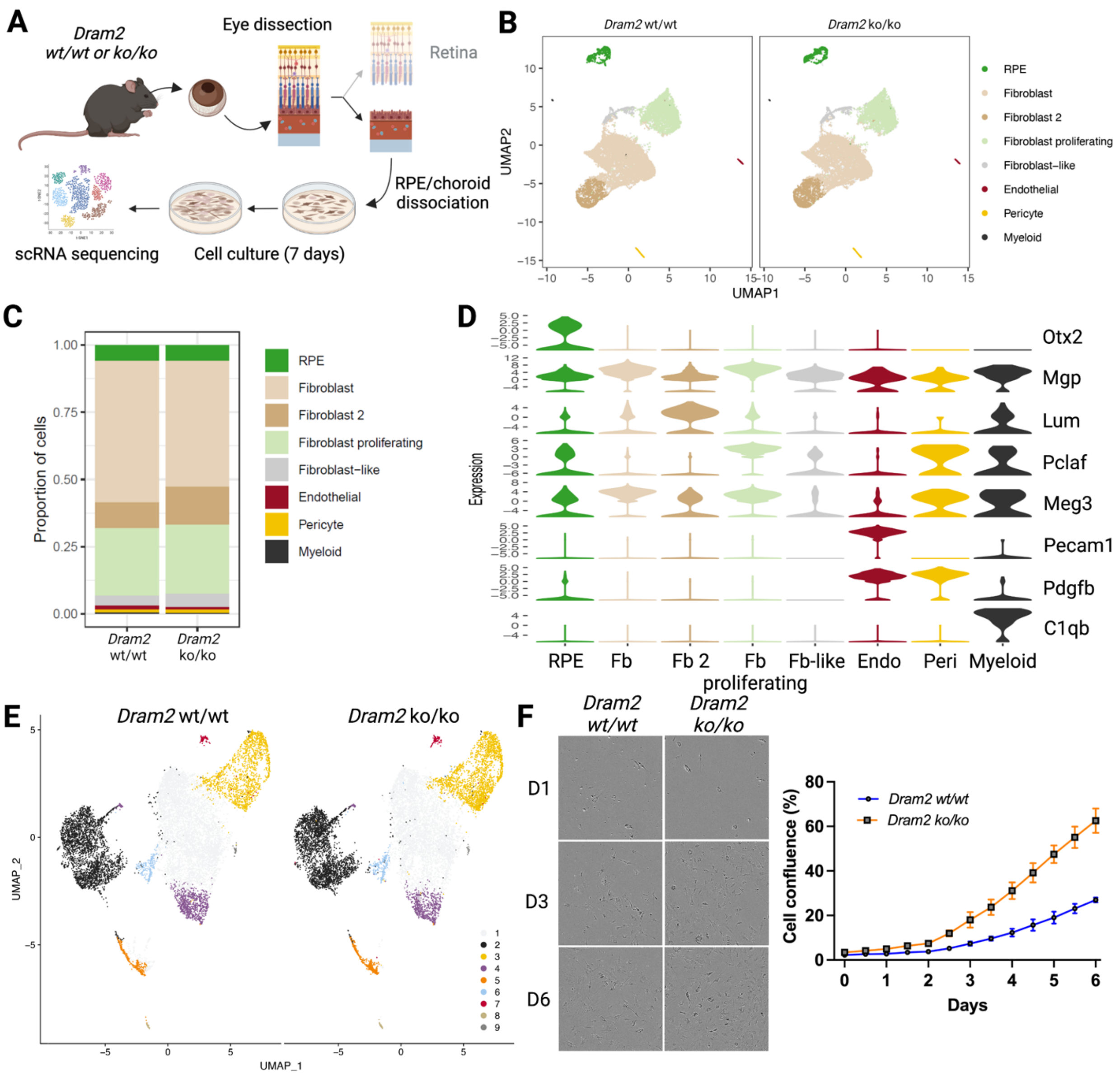

Figure S2: Dram2 loss increases proliferation of choroidal cells. (A). Experimental design of the single cell RNA sequencing (scRNAseq) analysis of the retinal pigment epithelium (RPE) and choroid samples from *Dram2* wt/wt and ko/ko mice. (B). UMAP representation of the RPE/choroid scRNA seq analysis from *Dram2* wt/wt and ko/ko mice, showing the main cell type clusters. (C). Proportion of the different cell types from identified clusters from the scRNAseq in the different genotypes. (D). Violin plot of key marker genes of the different identified clusters (RPE, retinal pigment epithelium; Fb, fibroblast; Fb 2, fibroblast 2; Fb proliferation, fibroblast proliferating; Fb-like, fibroblast-like; Endo, endothelial, Peri, pericyte). (E). UMAP representation of the sub-clustering of Fb, Fb2, Fb proliferating, Fb-like clusters showing no difference between *Dram2* wt/wt and ko/ko samples. (F). Representative bright field images of *Dram2* wt/wt and ko/ko RPE/choroid cells after 1 day (D1), 3 days (D3) and 6 days (D6) in culture (left) and corresponding proliferation curves (right) representing cell confluence (%) over time (n=4 eyes per well).

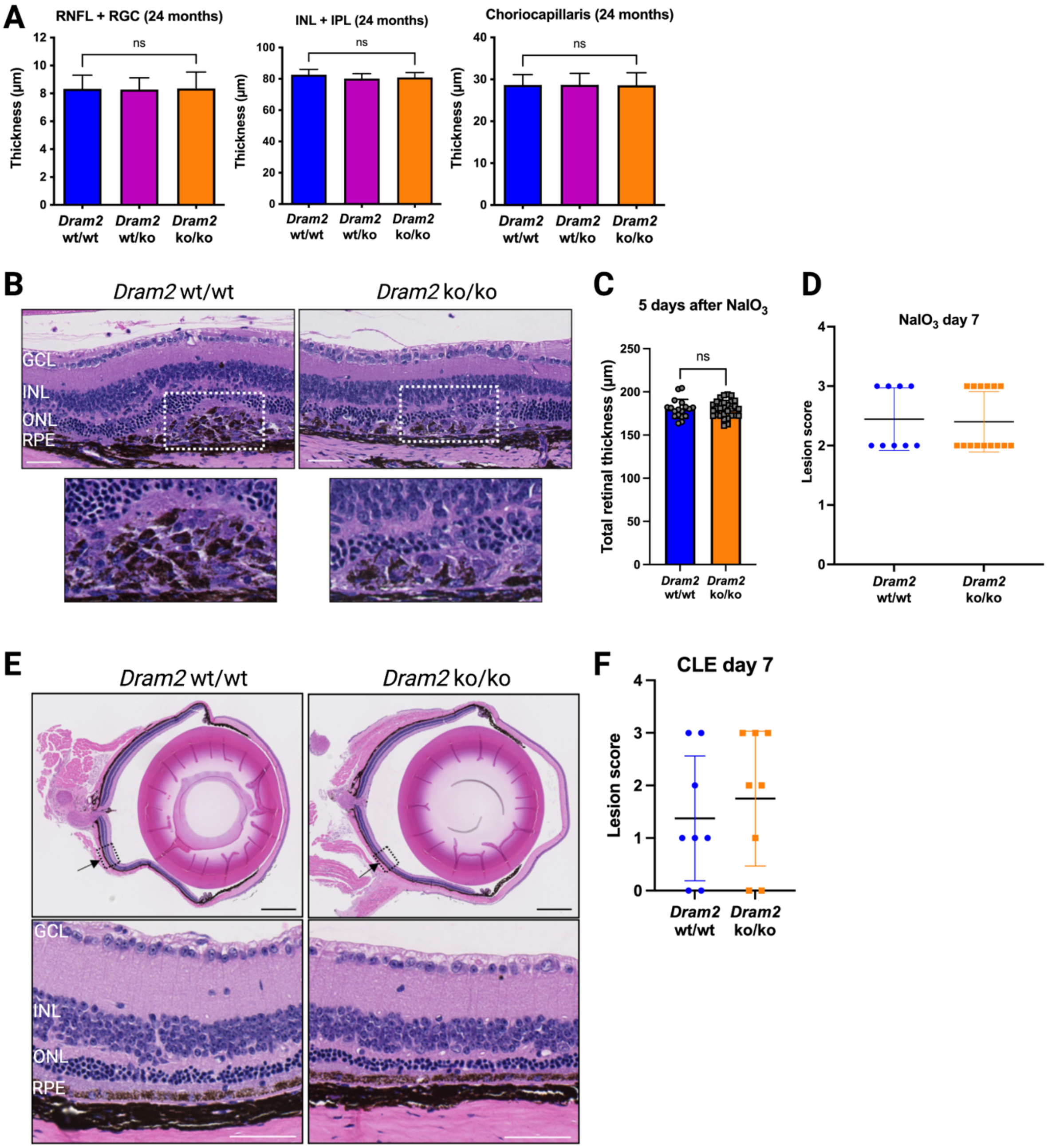

Figure S3: Additional *in vivo* analysis showing absence of phenotype in mice lacking Dram2. (A) Quantification of the different retinal layers thickness in *Dram2* wt/wt, wt/ko, and ko/ko mice at 24 months (RNFL, retina nerve fiber layer; RGC, retinal ganglion cells, INL, inner nuclear layer; IPL, inner plexiform layer; ns, not significant). (B) Histopathological analysis of *Dram2* wt/wt (left) and ko/ko (right) retinas after $NaIO_3$ treatment (day 7). Scale bar = 50 µm. (C) Retinal thickness measured by OCT in *Dram2* wt/wt and ko/ko mice 5 days after $NaIO_3$ injection. Unpaired t test; ns, not significant. (D) Lesion scores of *Dram2* wt/wt and ko/ko after $NaIO_3$ (day 7) in *Dram2* wt/wt and *Dram2* ko/ko retinas. (E) Histopathological analysis of *Dram2* wt/wt and ko/ko retinas after CLE (day 7). Scale bar: black = 500 µm, white = 50 µm. (F). Lesion scores of *Dram2* wt/wt and ko/ko after CLE exposure. GCL, ganglion cell layer; INL, inner nuclear layer; ONL, outer nuclear layer; RPE, retinal pigment epithelium.

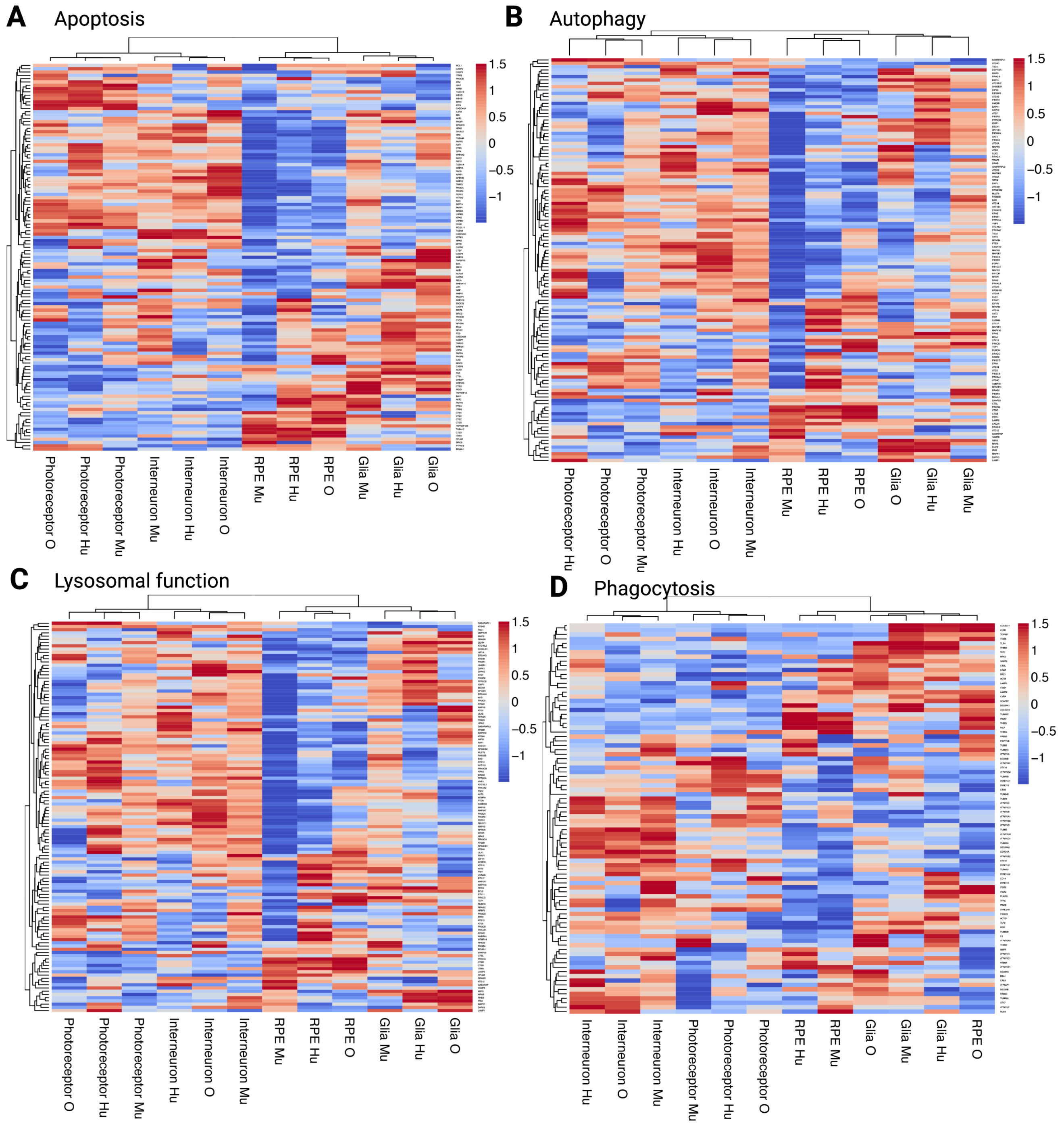

Figure S4: Heatmaps showing expression of marker genes for different cellular biological processes by cell types and models (A) Heatmap of the unsupervised hierarchical cluster analysis of top Apoptosis marker genes per cell types identified in all three datasets. (B) Heatmap of the unsupervised hierarchical cluster analysis of top Autophagy marker genes per cell types identified in all three datasets. (C) Heatmap of the unsupervised hierarchical cluster analysis of top Lysosomal function marker genes per cell types identified in all three datasets. (D) Heatmap of the unsupervised hierarchical cluster analysis of top Phagocytosis marker genes per cell types identified in all three datasets. Mu, mouse retina scRNAseq; O, hPSC- retinal organoid scRNAseq; Hu, human snRNAseq.
